# Supplementary figures and images for: Palmitoylated importin α regulates mitotic spindle orientation through interaction with NuMA
Source: EMBO Rep. 2025 May 27;26(13):3280–304. doi: 10.1038/s44319-025-00484-8 (PMC12238373; doi:10.1038/s44319-025-00484-8)

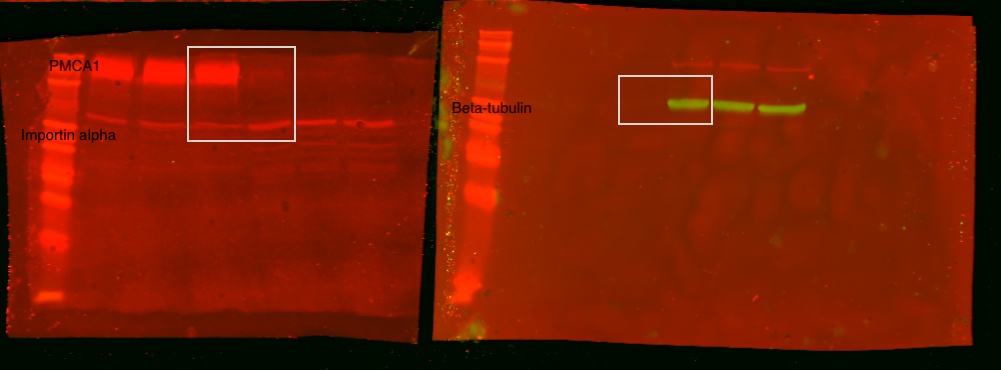

Supplement: Supplementary file 2 — Source data Fig. 1 [file 44319_2025_484_MOESM2_ESM.zip › Figure 1 Raw Data/1E/Figure 1E Western blot.png]

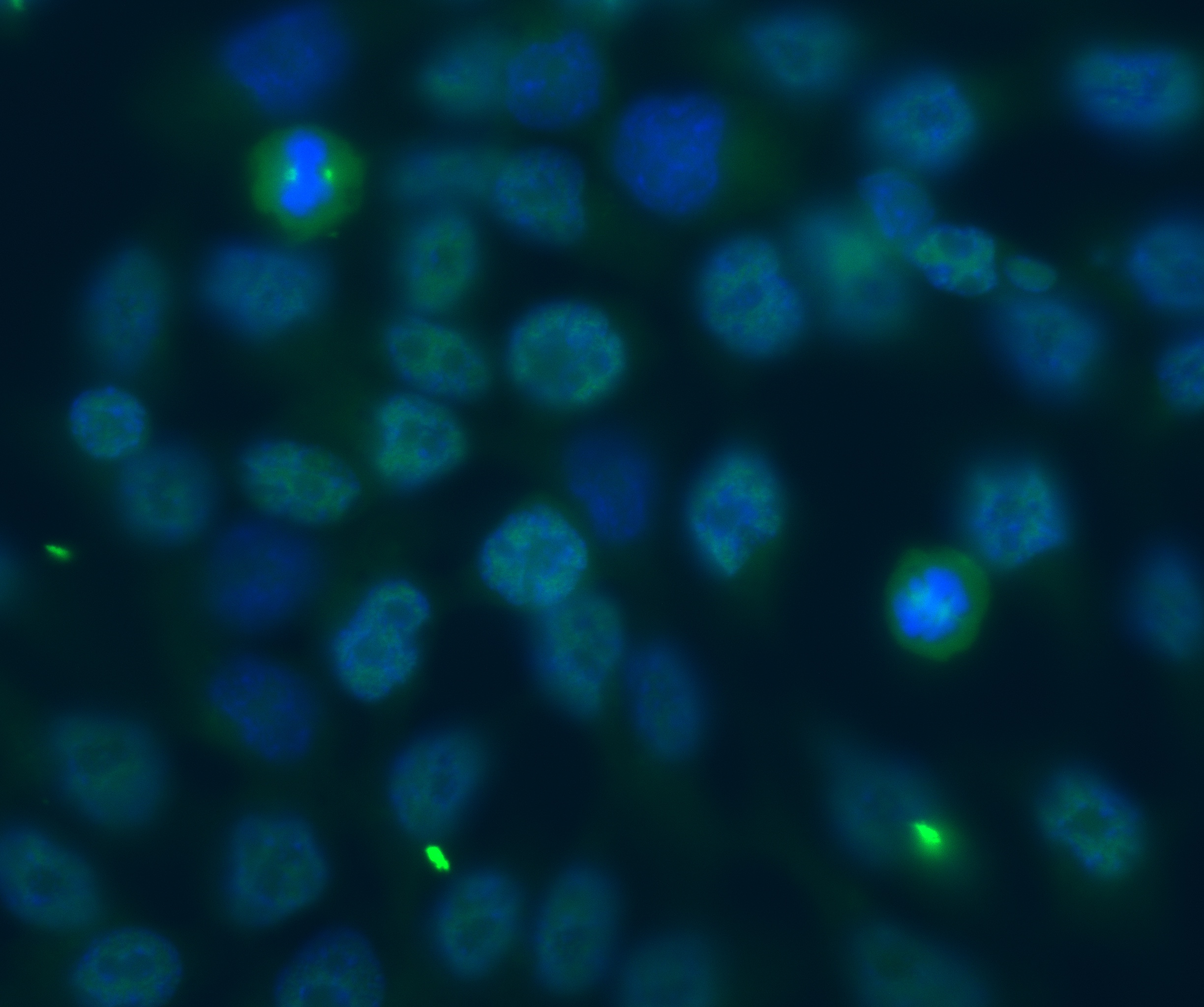

Supplement: Supplementary file 2 — Source data Fig. 1 [file 44319_2025_484_MOESM2_ESM.zip › Figure 1 Raw Data/1G/Figure 1G DMSO representative image.jpg]

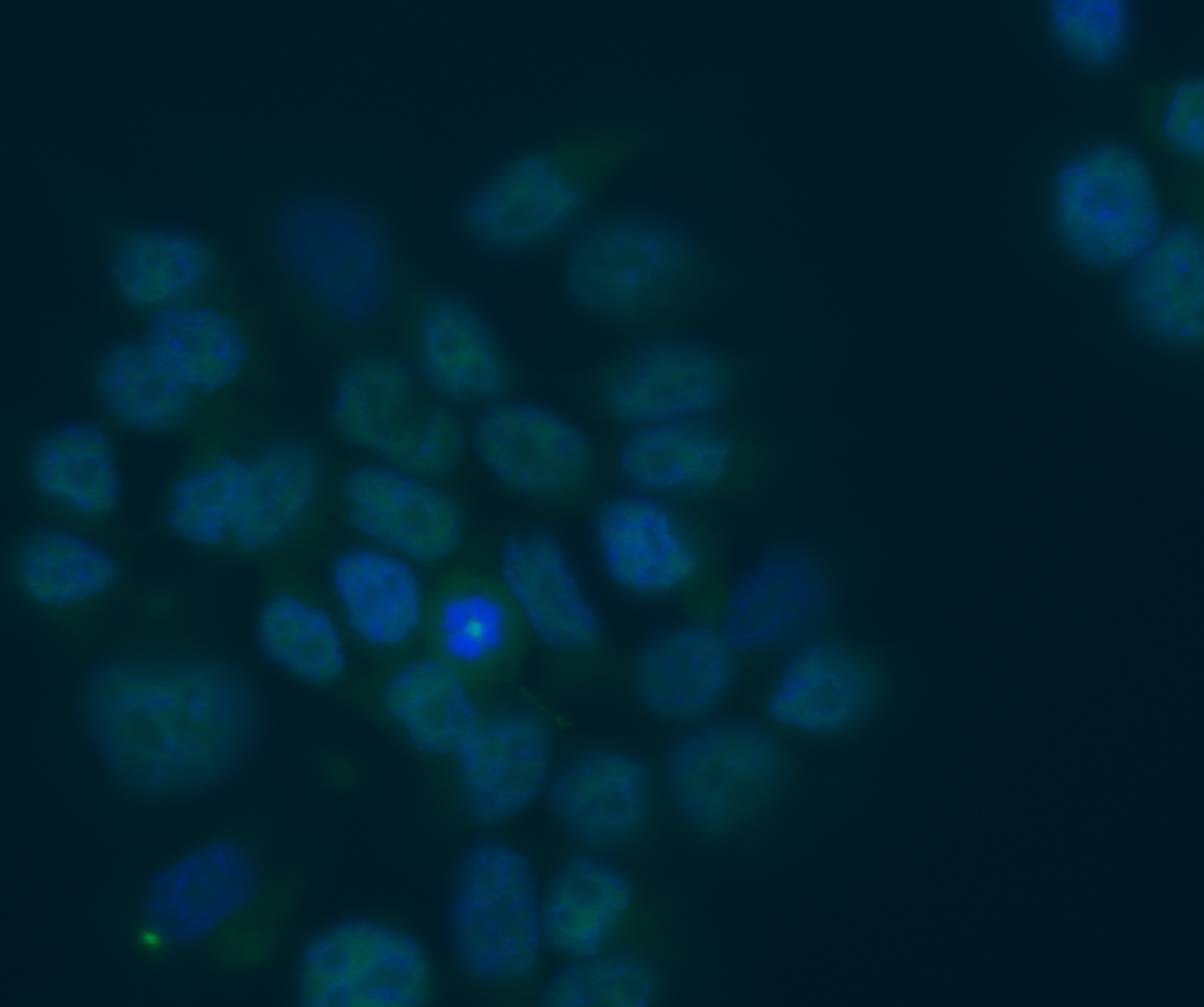

Supplement: Supplementary file 2 — Source data Fig. 1 [file 44319_2025_484_MOESM2_ESM.zip › Figure 1 Raw Data/1G/Figure 1G Wnt-C59 Representative image.jpg]

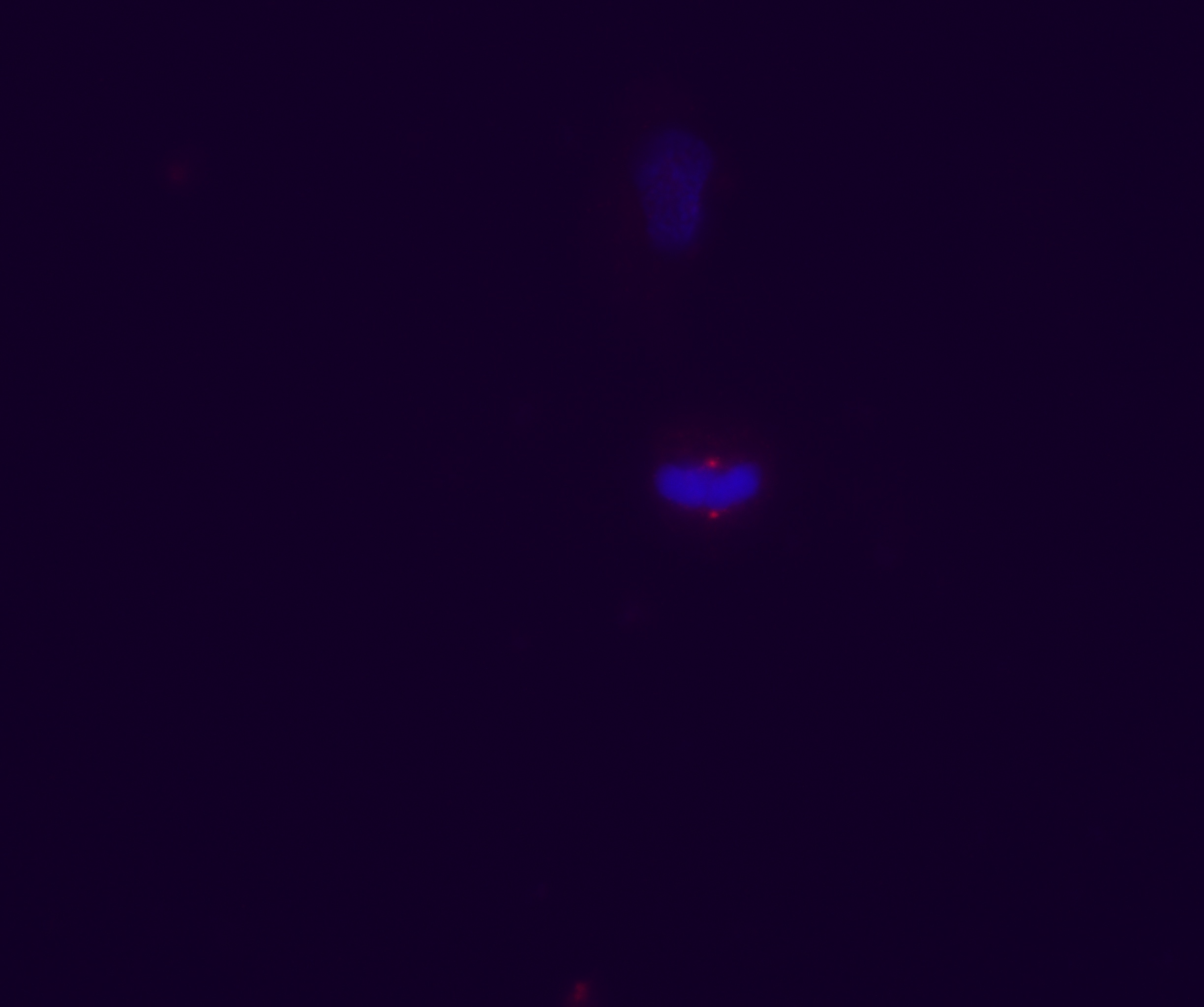

Supplement: Supplementary file 2 — Source data Fig. 1 [file 44319_2025_484_MOESM2_ESM.zip › Figure 1 Raw Data/1K/Figure 1K DMSO representative image.jpg]

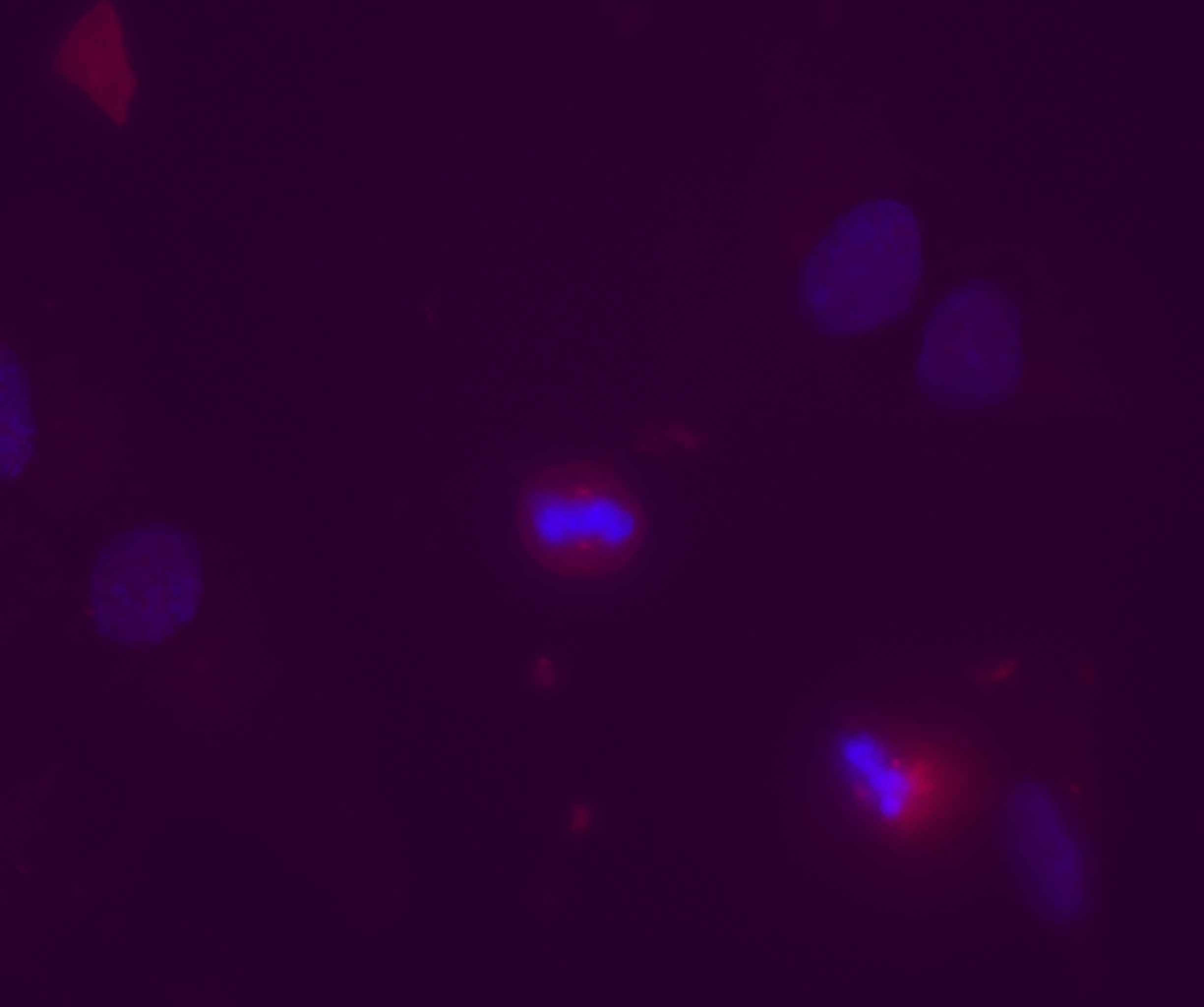

Supplement: Supplementary file 2 — Source data Fig. 1 [file 44319_2025_484_MOESM2_ESM.zip › Figure 1 Raw Data/1K/Figure 1K Palmostatin representative image.jpg]

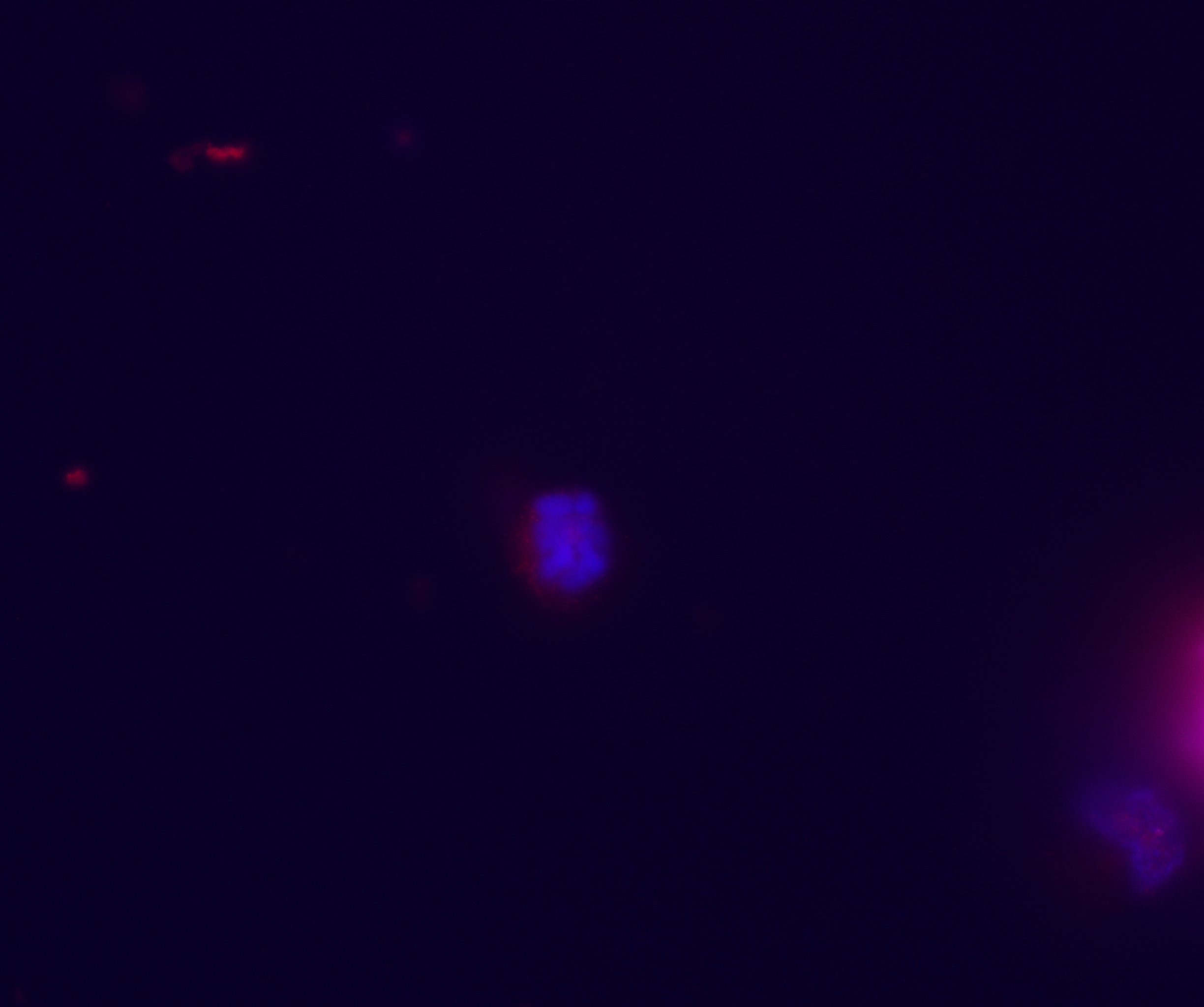

Supplement: Supplementary file 2 — Source data Fig. 1 [file 44319_2025_484_MOESM2_ESM.zip › Figure 1 Raw Data/1K/Figure 1K Wnt-C59 representative image.jpg]

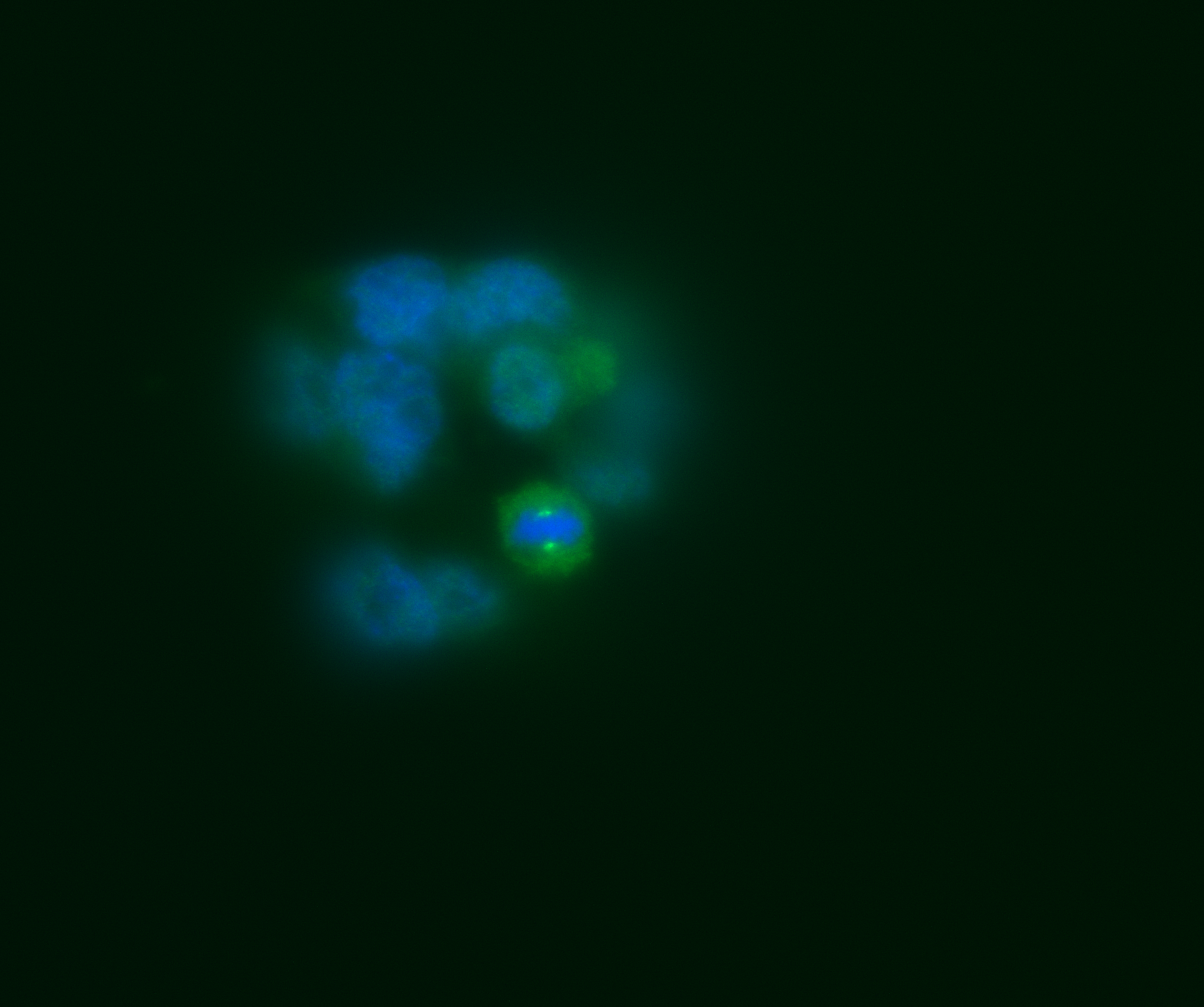

Supplement: Supplementary file 2 — Source data Fig. 1 [file 44319_2025_484_MOESM2_ESM.zip › Figure 1 Raw Data/1I/Figure 1I DMSO Representative image.png]

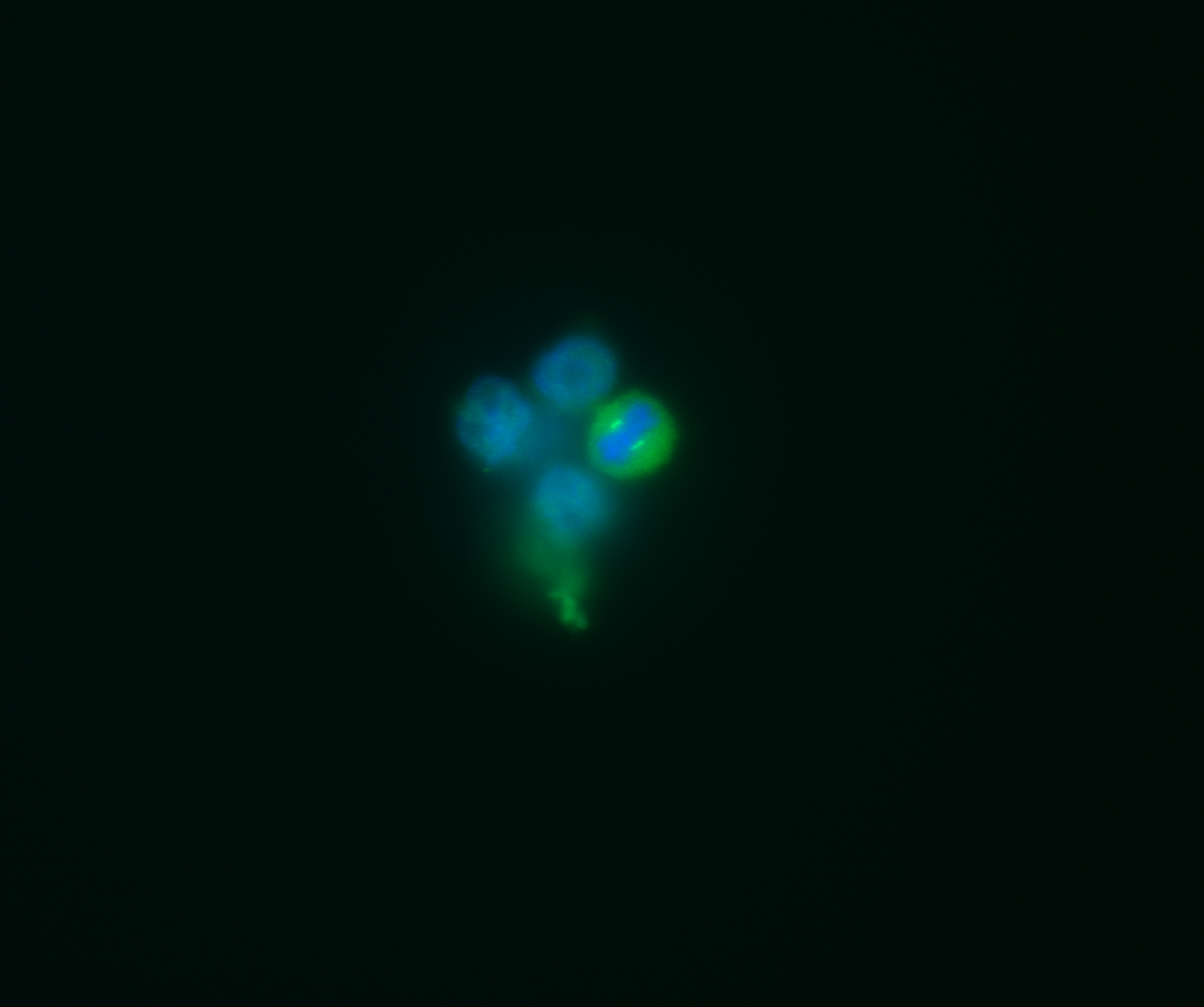

Supplement: Supplementary file 2 — Source data Fig. 1 [file 44319_2025_484_MOESM2_ESM.zip › Figure 1 Raw Data/1I/Figure 1I Wnt-C59 Representative image.png]

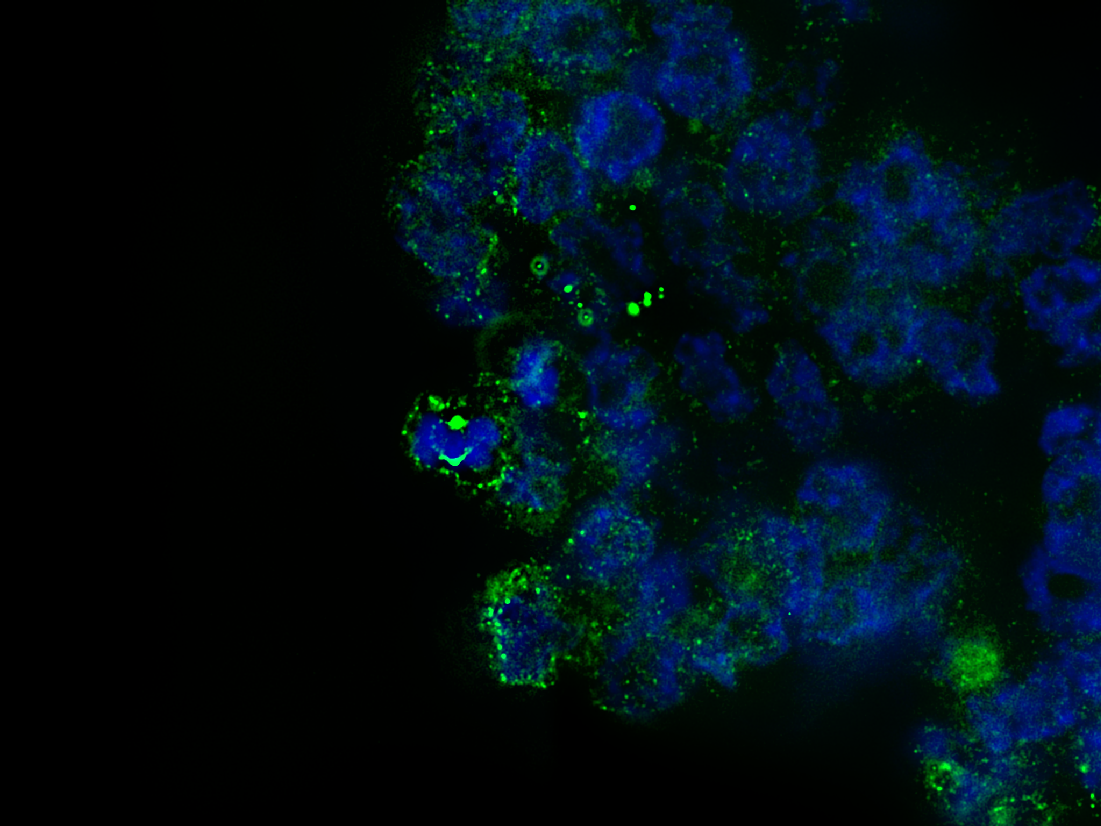

Supplement: Supplementary file 2 — Source data Fig. 1 [file 44319_2025_484_MOESM2_ESM.zip › Figure 1 Raw Data/1A/Figure 1A DMSO Rep.TIF]

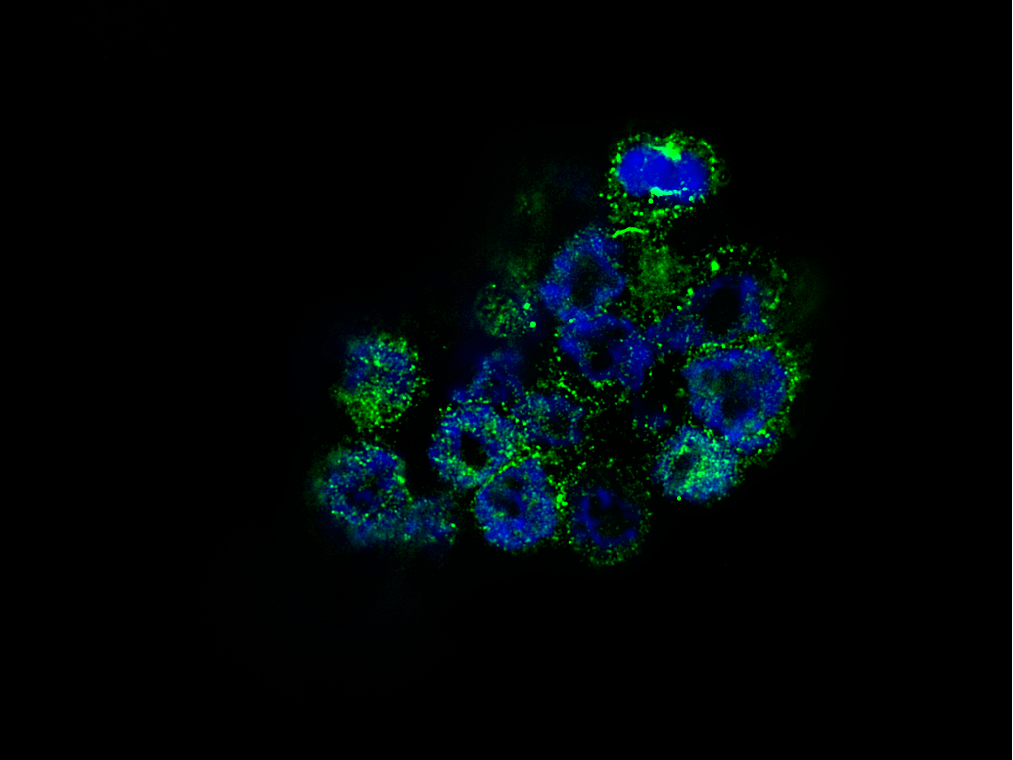

Supplement: Supplementary file 2 — Source data Fig. 1 [file 44319_2025_484_MOESM2_ESM.zip › Figure 1 Raw Data/1A/Figure 1A Importazole Rep.TIF]

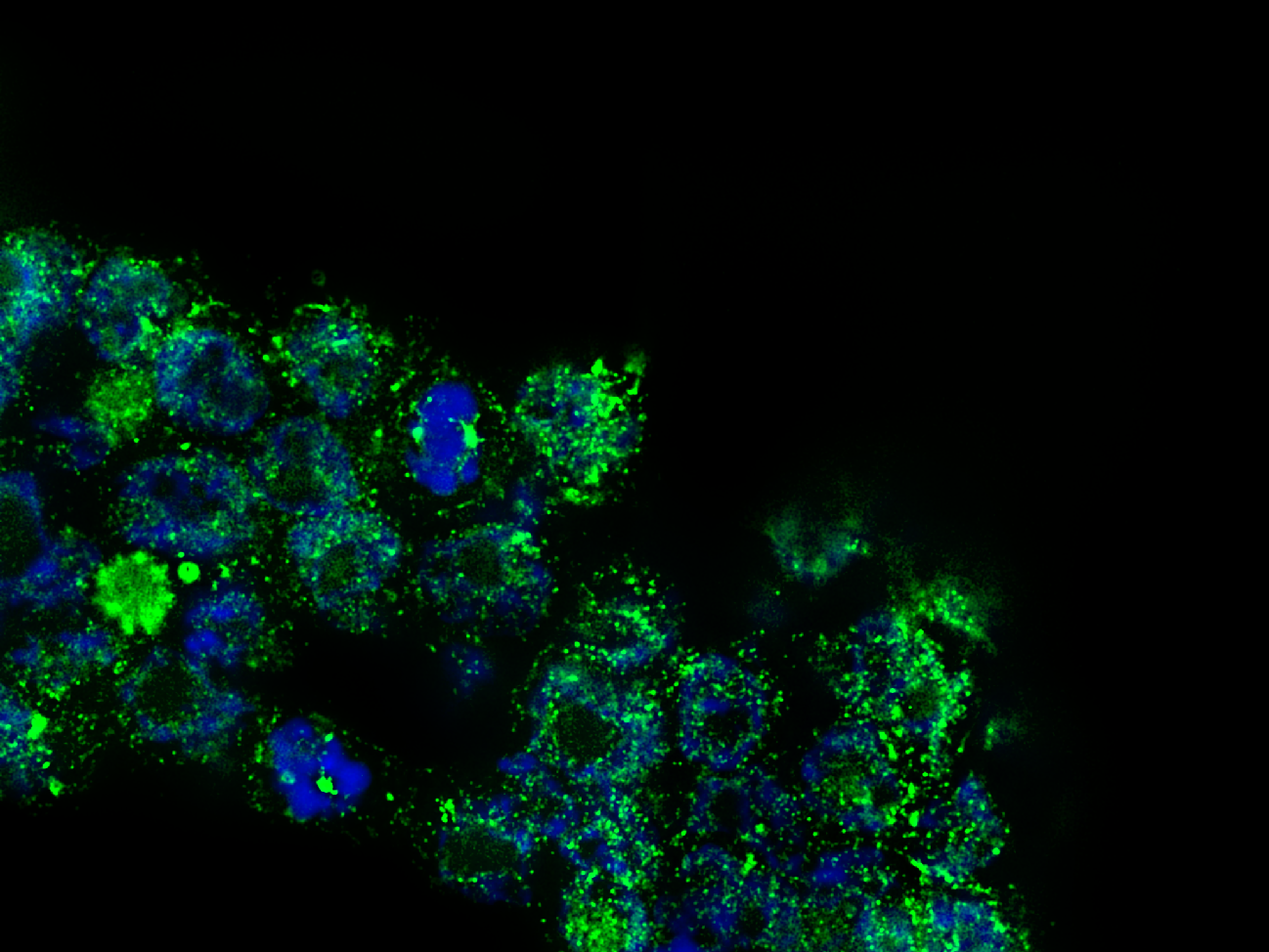

Supplement: Supplementary file 2 — Source data Fig. 1 [file 44319_2025_484_MOESM2_ESM.zip › Figure 1 Raw Data/1A/Figure 1A Ivermectin Rep.TIF]

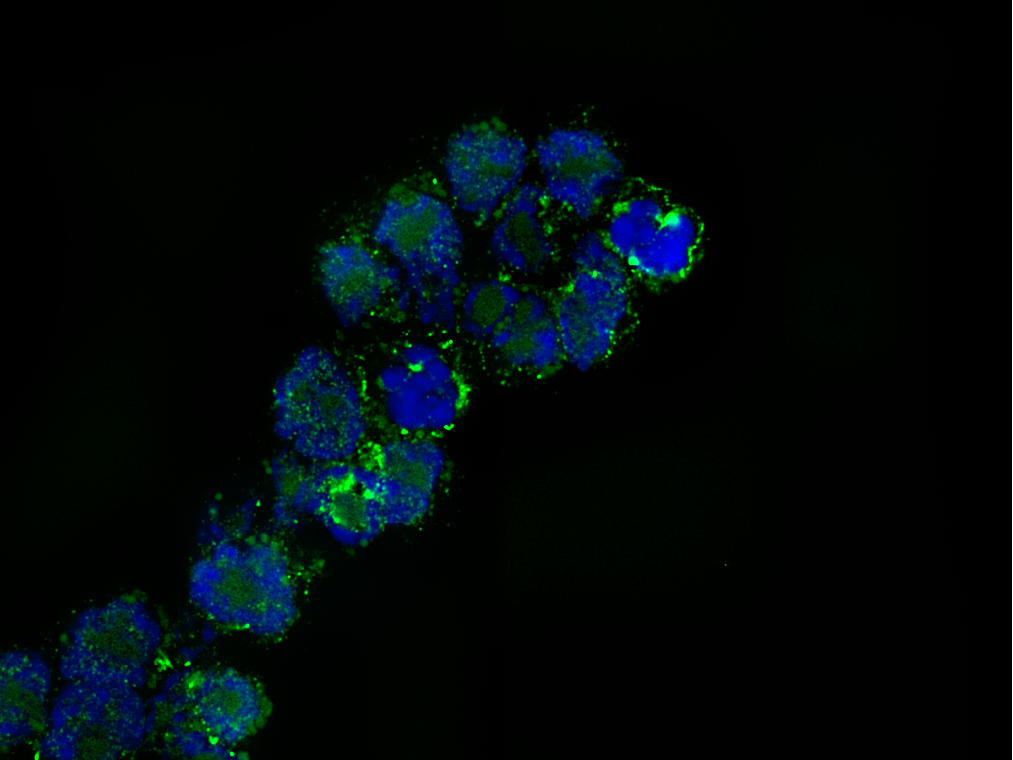

Supplement: Supplementary file 2 — Source data Fig. 1 [file 44319_2025_484_MOESM2_ESM.zip › Figure 1 Raw Data/1A/Figure 1A Palmostatin Rep.TIF]

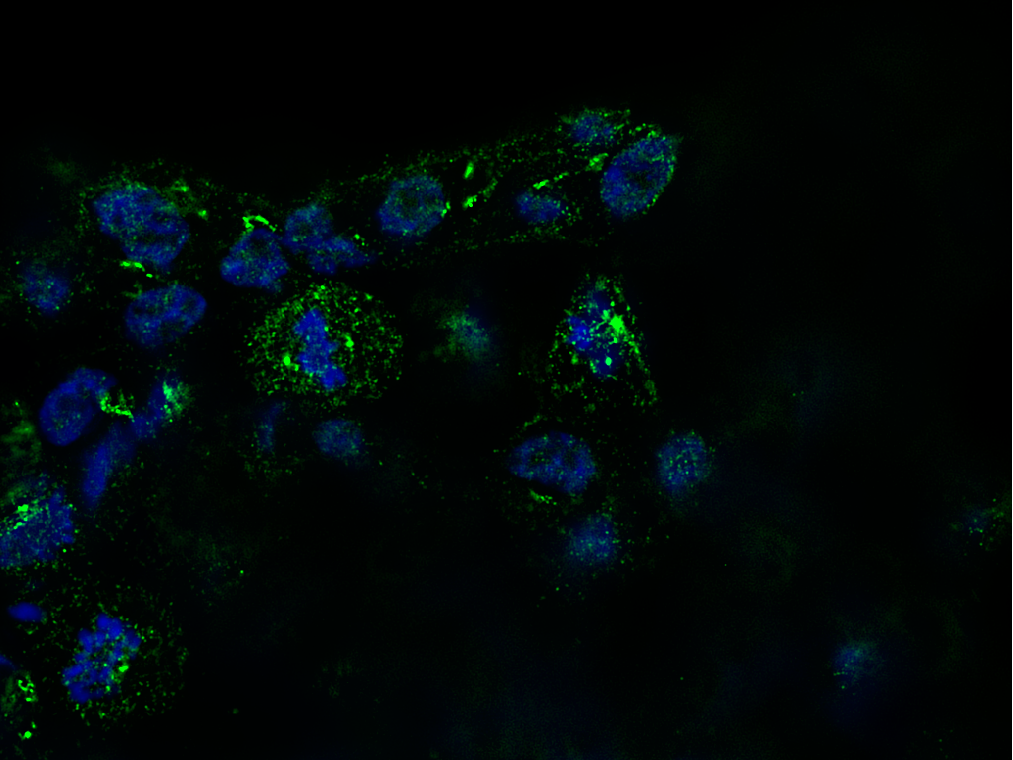

Supplement: Supplementary file 2 — Source data Fig. 1 [file 44319_2025_484_MOESM2_ESM.zip › Figure 1 Raw Data/1A/Figure 1A Wnt-C59 Rep.TIF]

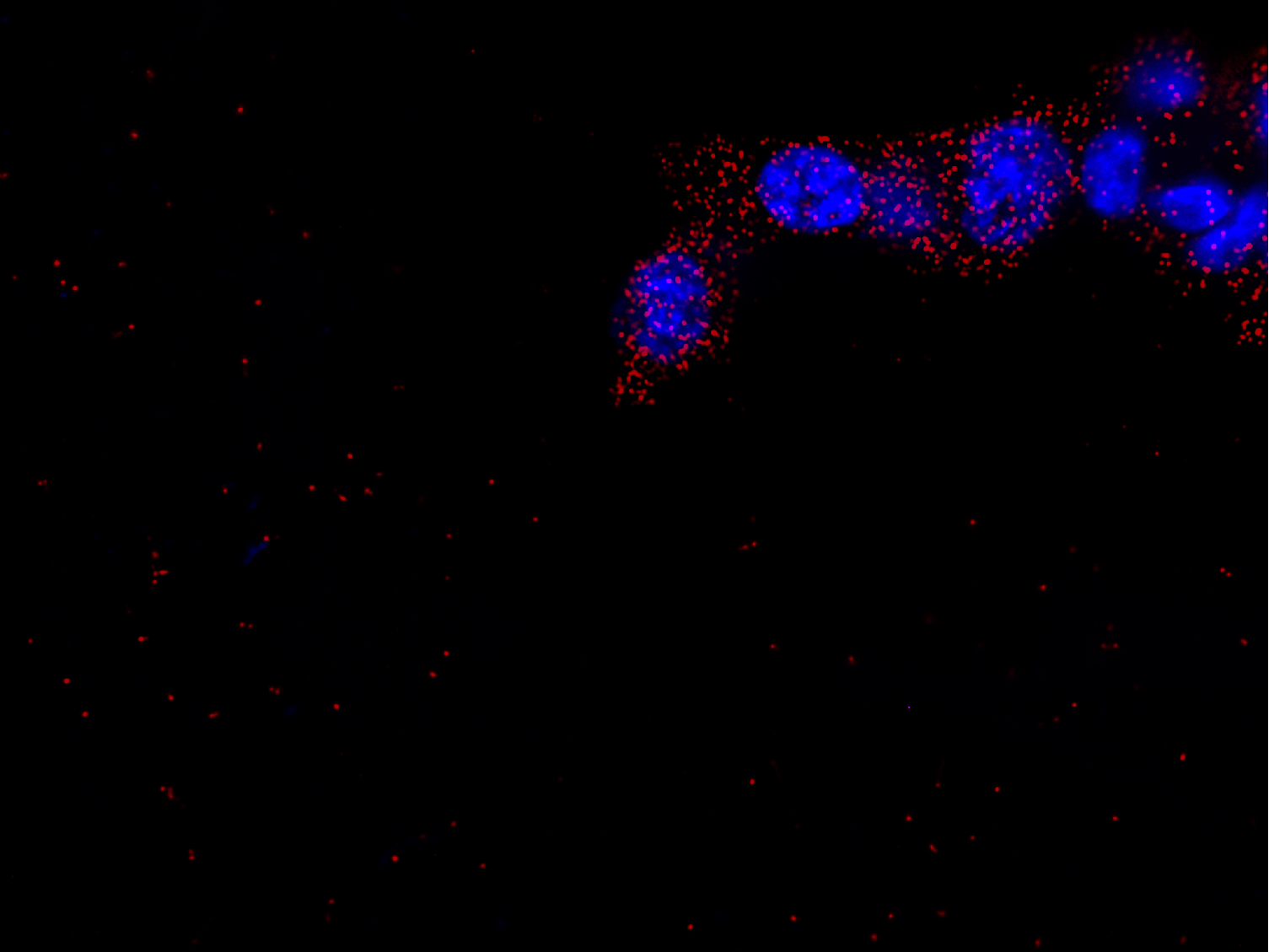

Supplement: Supplementary file 3 — Source data Fig. 2 [file 44319_2025_484_MOESM3_ESM.zip › Figure 2 Raw Data/2E/Figure 2E Dlg PLA Palmostatin HCT116 Interphase Representative Image.TIF]

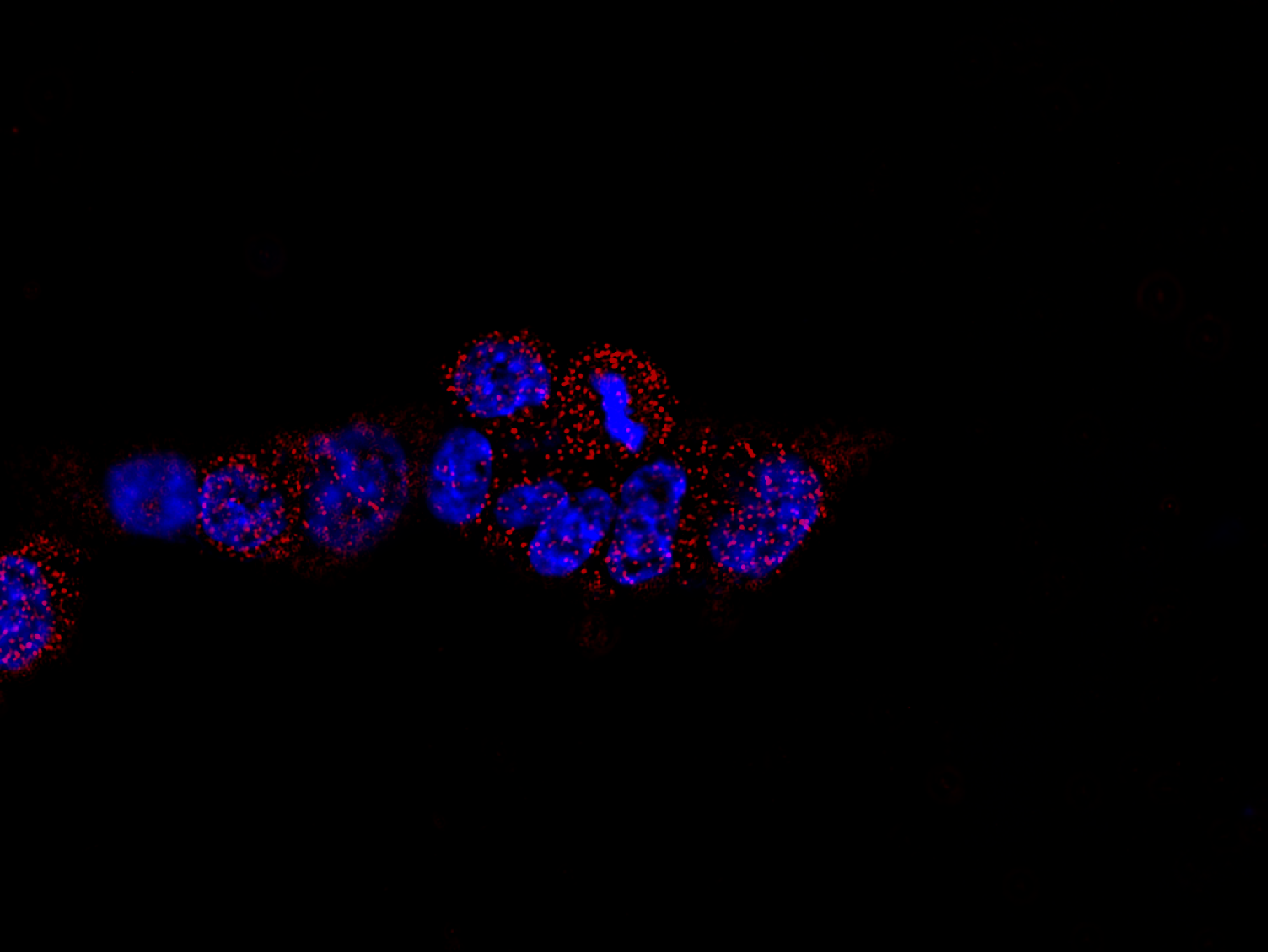

Supplement: Supplementary file 3 — Source data Fig. 2 [file 44319_2025_484_MOESM3_ESM.zip › Figure 2 Raw Data/2E/Figure 2E Dlg PLA Palmostatin HCT116 Metaphase Representative Image.TIF]

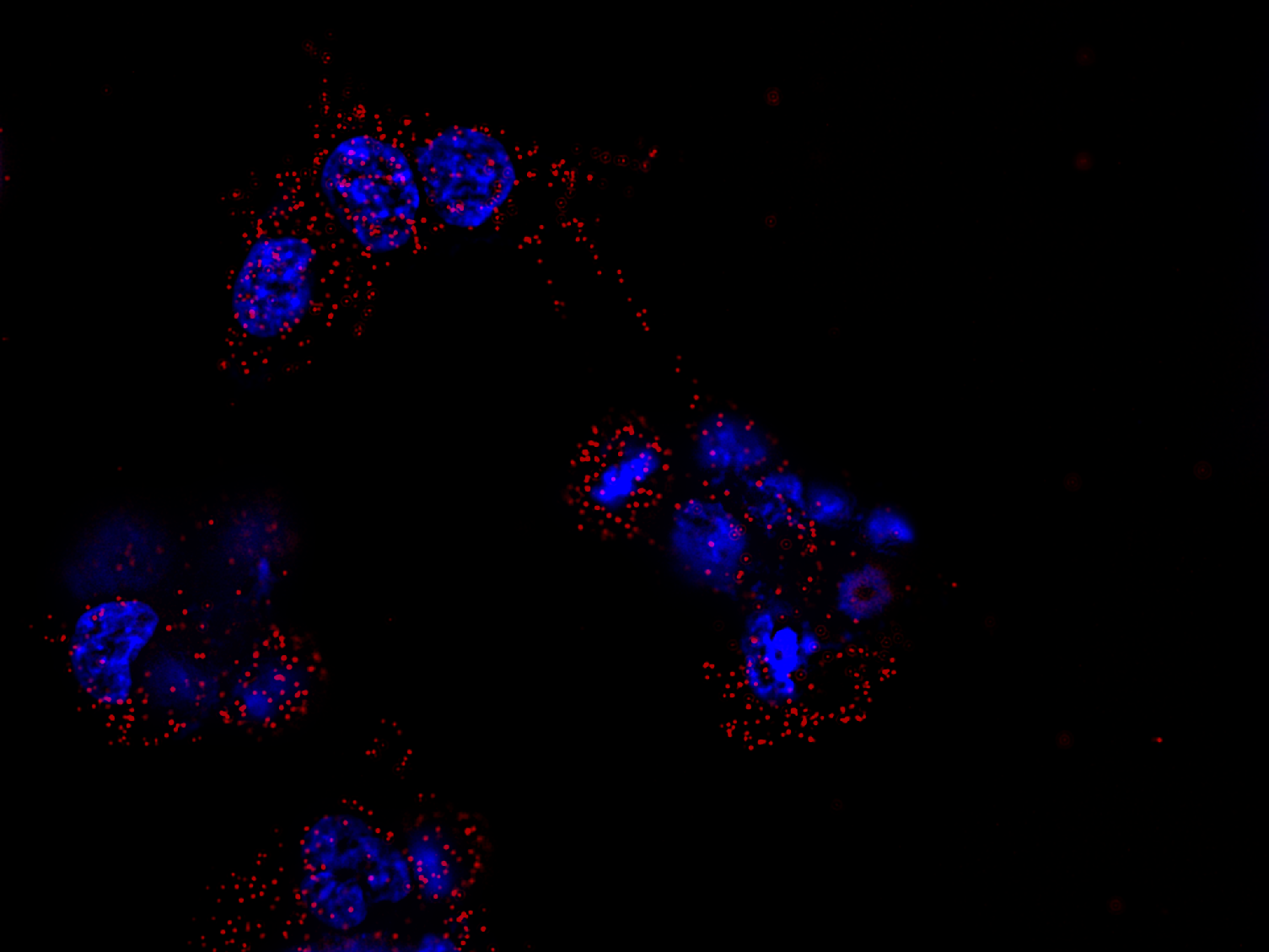

Supplement: Supplementary file 3 — Source data Fig. 2 [file 44319_2025_484_MOESM3_ESM.zip › Figure 2 Raw Data/2E/Figure 2E Dlg PLA Wnt-C59 HCT116 Interphase Representative Image.TIF]

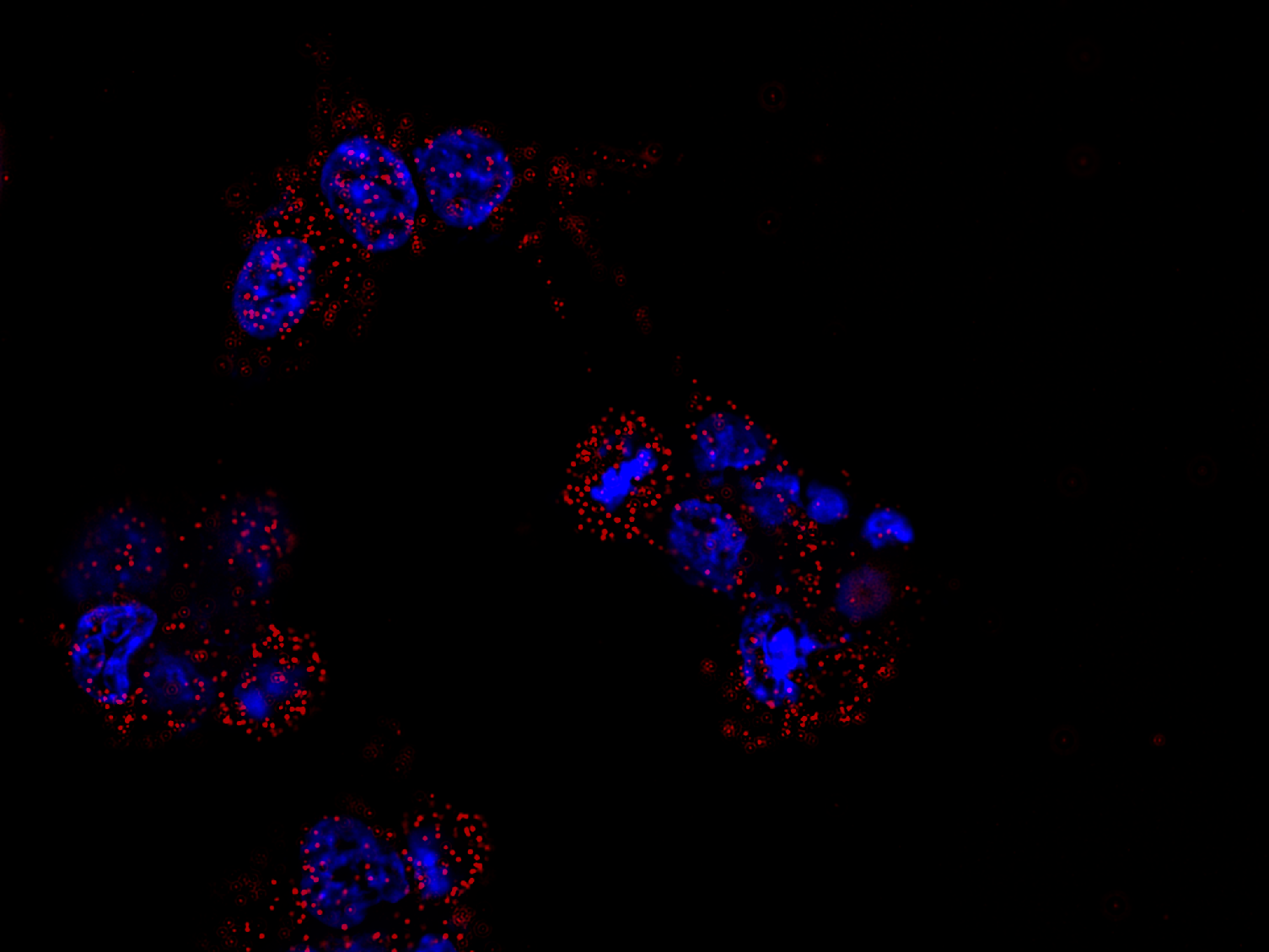

Supplement: Supplementary file 3 — Source data Fig. 2 [file 44319_2025_484_MOESM3_ESM.zip › Figure 2 Raw Data/2E/Figure 2E Dlg PLA Wnt-C59 HCT116 Metaphase Representative Image.TIF]

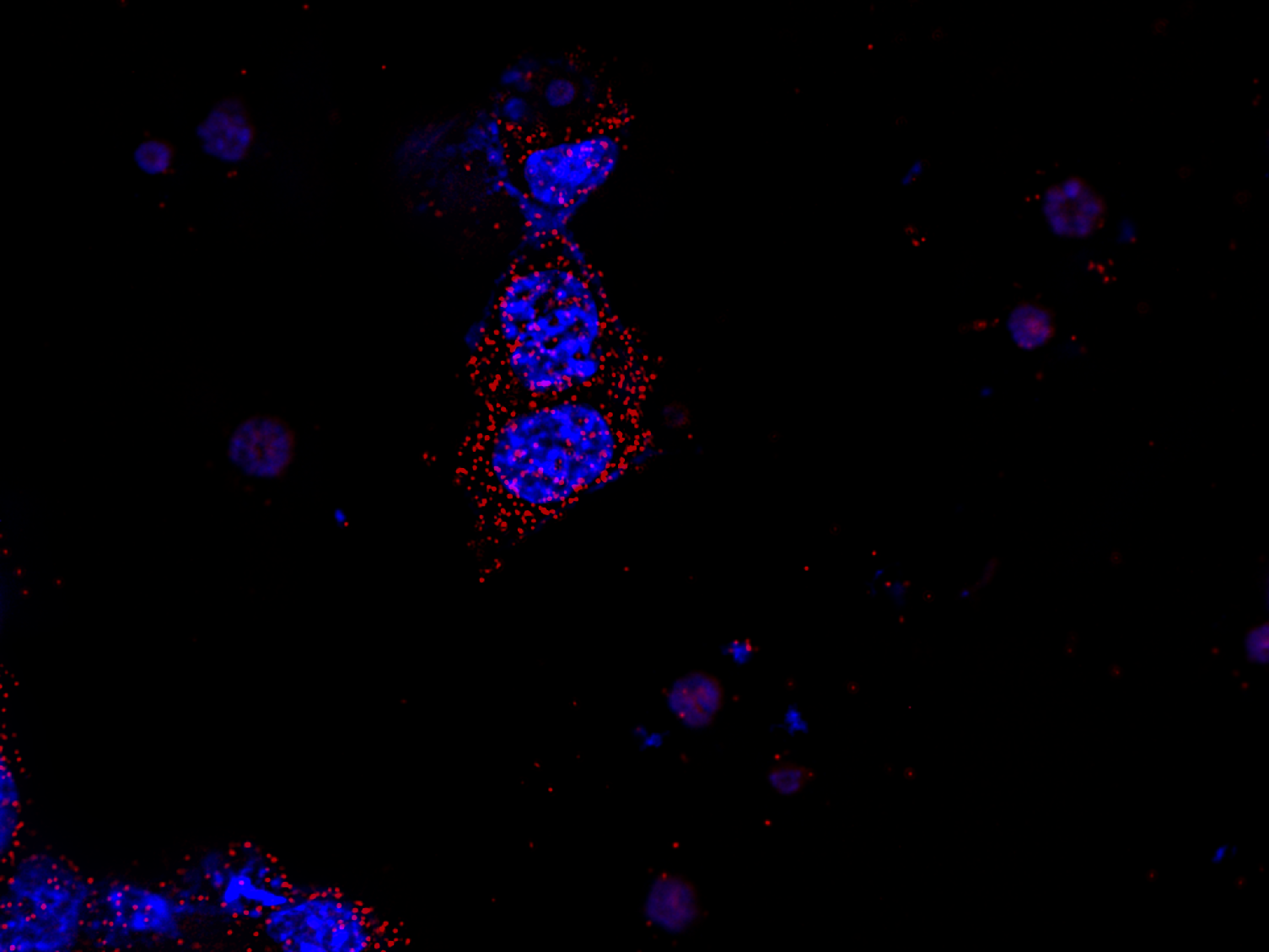

Supplement: Supplementary file 3 — Source data Fig. 2 [file 44319_2025_484_MOESM3_ESM.zip › Figure 2 Raw Data/2E/Figure 2E Dlg PLA DMSO HCT116 Interphase Representative Image.TIF]

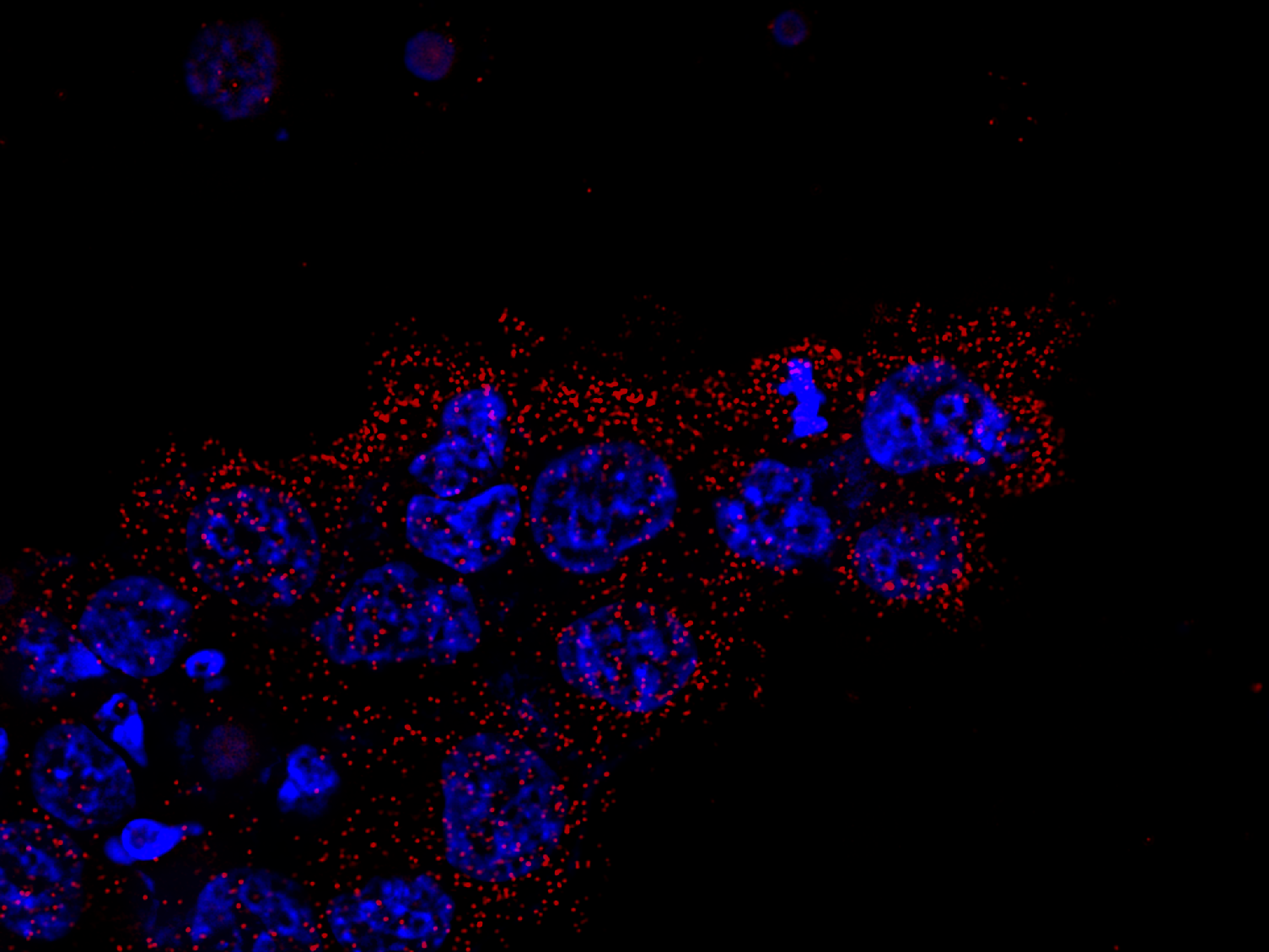

Supplement: Supplementary file 3 — Source data Fig. 2 [file 44319_2025_484_MOESM3_ESM.zip › Figure 2 Raw Data/2E/Figure 2E Dlg PLA DMSO HCT116 Metaphase Representative Image.TIF]

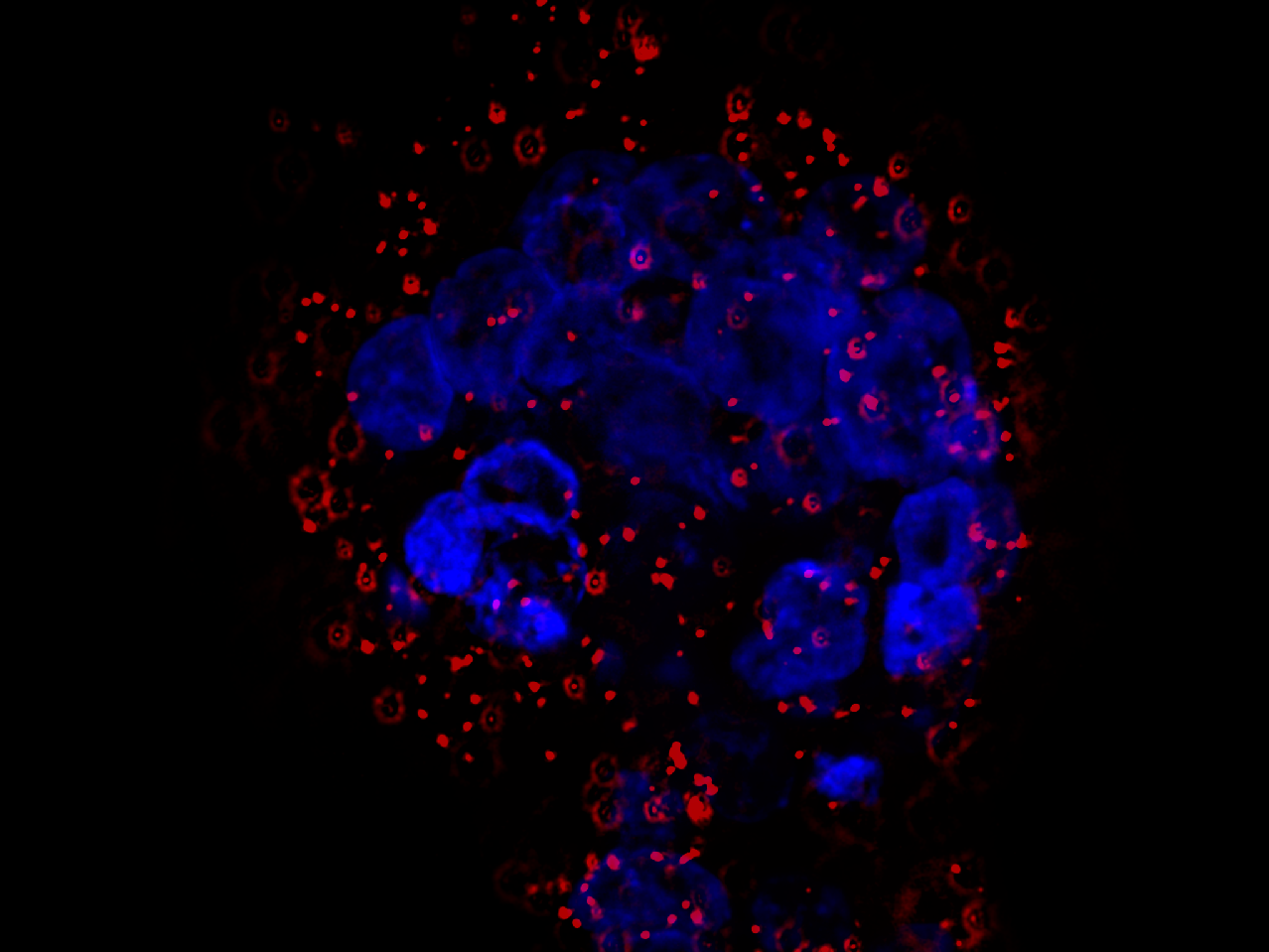

Supplement: Supplementary file 3 — Source data Fig. 2 [file 44319_2025_484_MOESM3_ESM.zip › Figure 2 Raw Data/2G/Figure 2G PLA rescue WT impa Wnt-C59 Representative image.png]

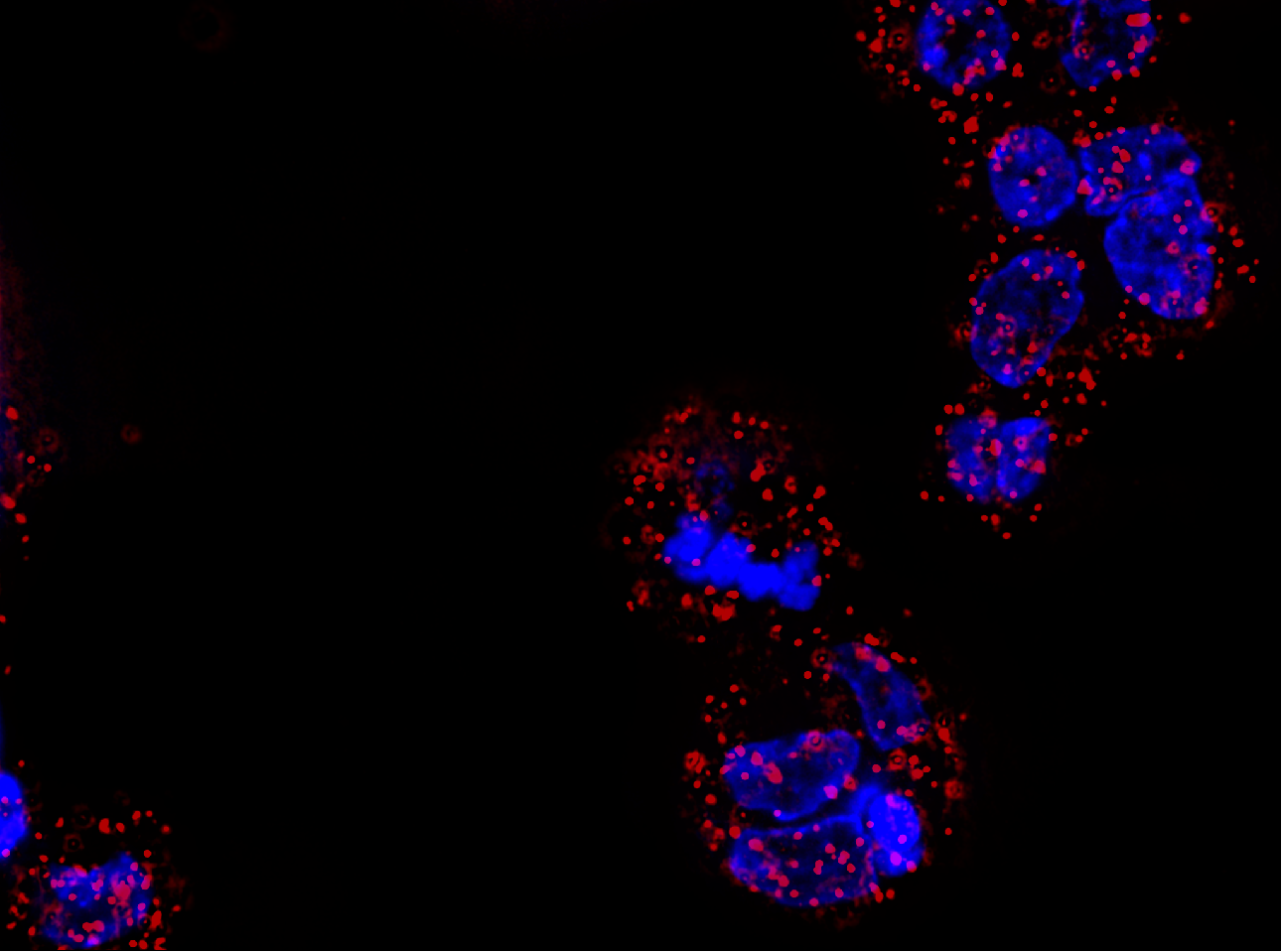

Supplement: Supplementary file 3 — Source data Fig. 2 [file 44319_2025_484_MOESM3_ESM.zip › Figure 2 Raw Data/2G/Figure 2G PLA Rescue impaCaax Wnt-C59 Representative Image.png]

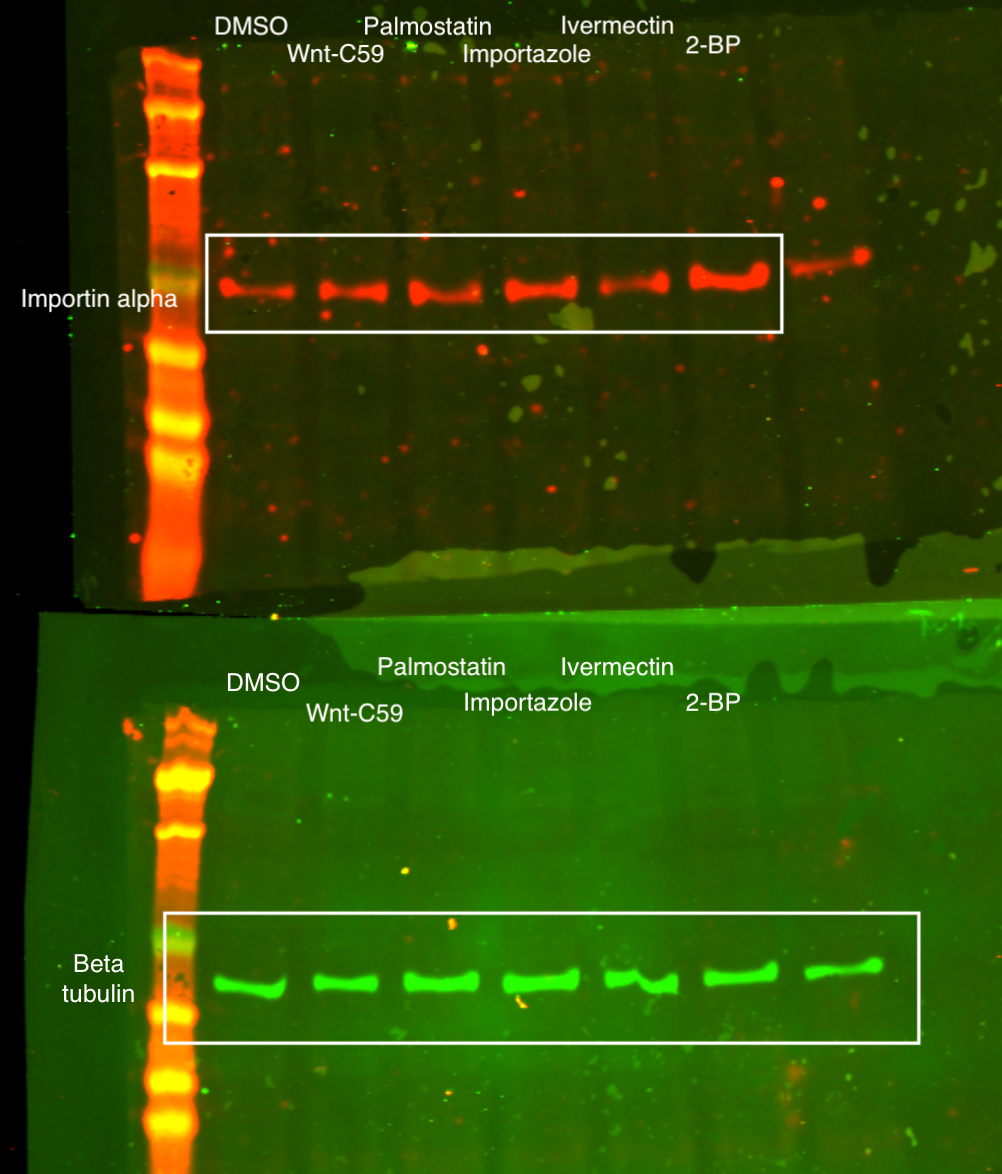

Supplement: Supplementary file 3 — Source data Fig. 2 [file 44319_2025_484_MOESM3_ESM.zip › Figure 2 Raw Data/2I/Figure 2I KPNA2 and E7 Expression Representative Blot.png]

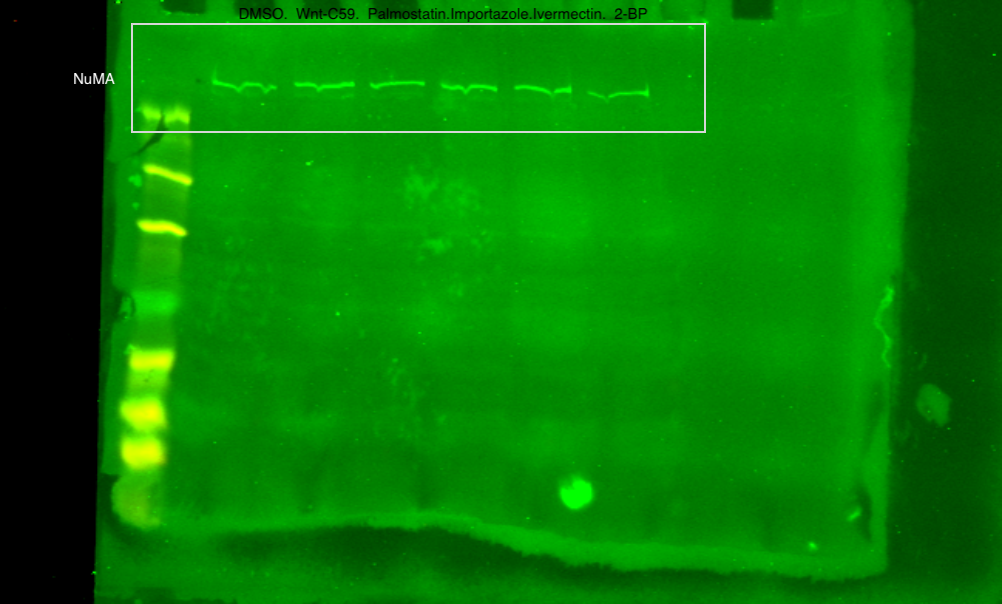

Supplement: Supplementary file 3 — Source data Fig. 2 [file 44319_2025_484_MOESM3_ESM.zip › Figure 2 Raw Data/2I/Figure 2I NuMA Expression Representative Blot.png]

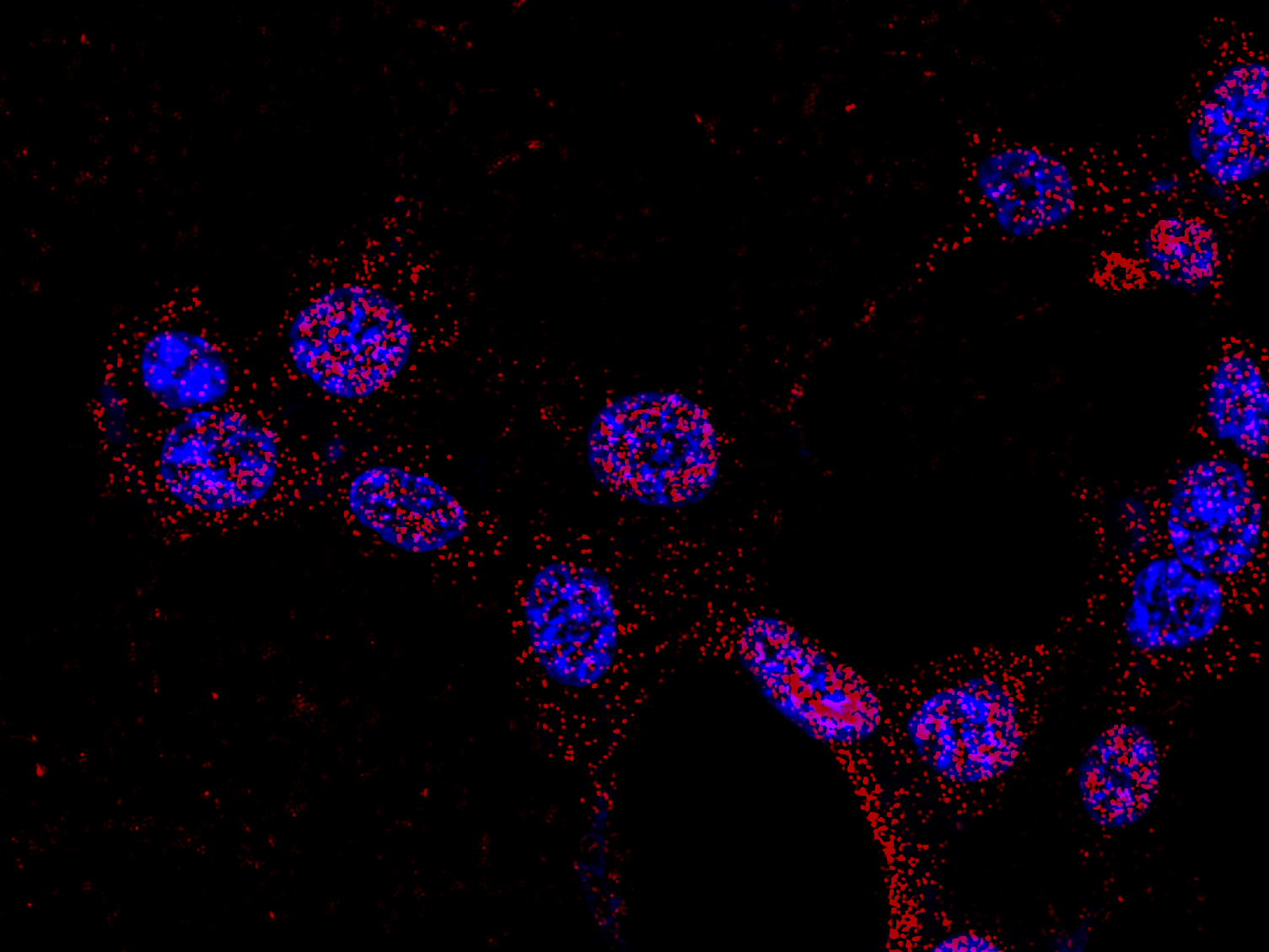

Supplement: Supplementary file 3 — Source data Fig. 2 [file 44319_2025_484_MOESM3_ESM.zip › Figure 2 Raw Data/2C/Figure 2C NuMA PLA DMSO HCT116 Interphase Representative Image.TIF]

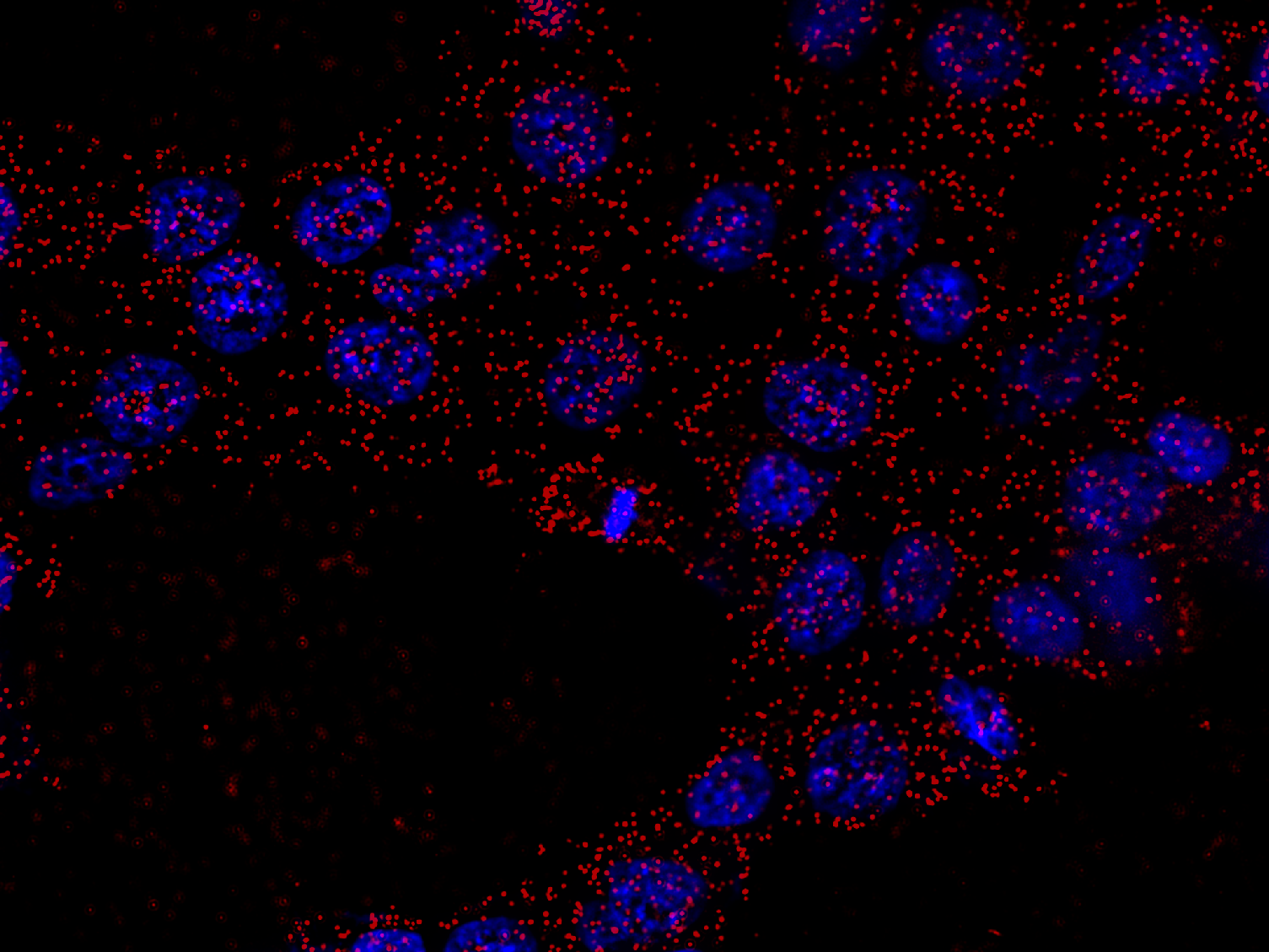

Supplement: Supplementary file 3 — Source data Fig. 2 [file 44319_2025_484_MOESM3_ESM.zip › Figure 2 Raw Data/2C/Figure 2C NuMA PLA DMSO HCT116 Mitotic Representative Image.TIF]

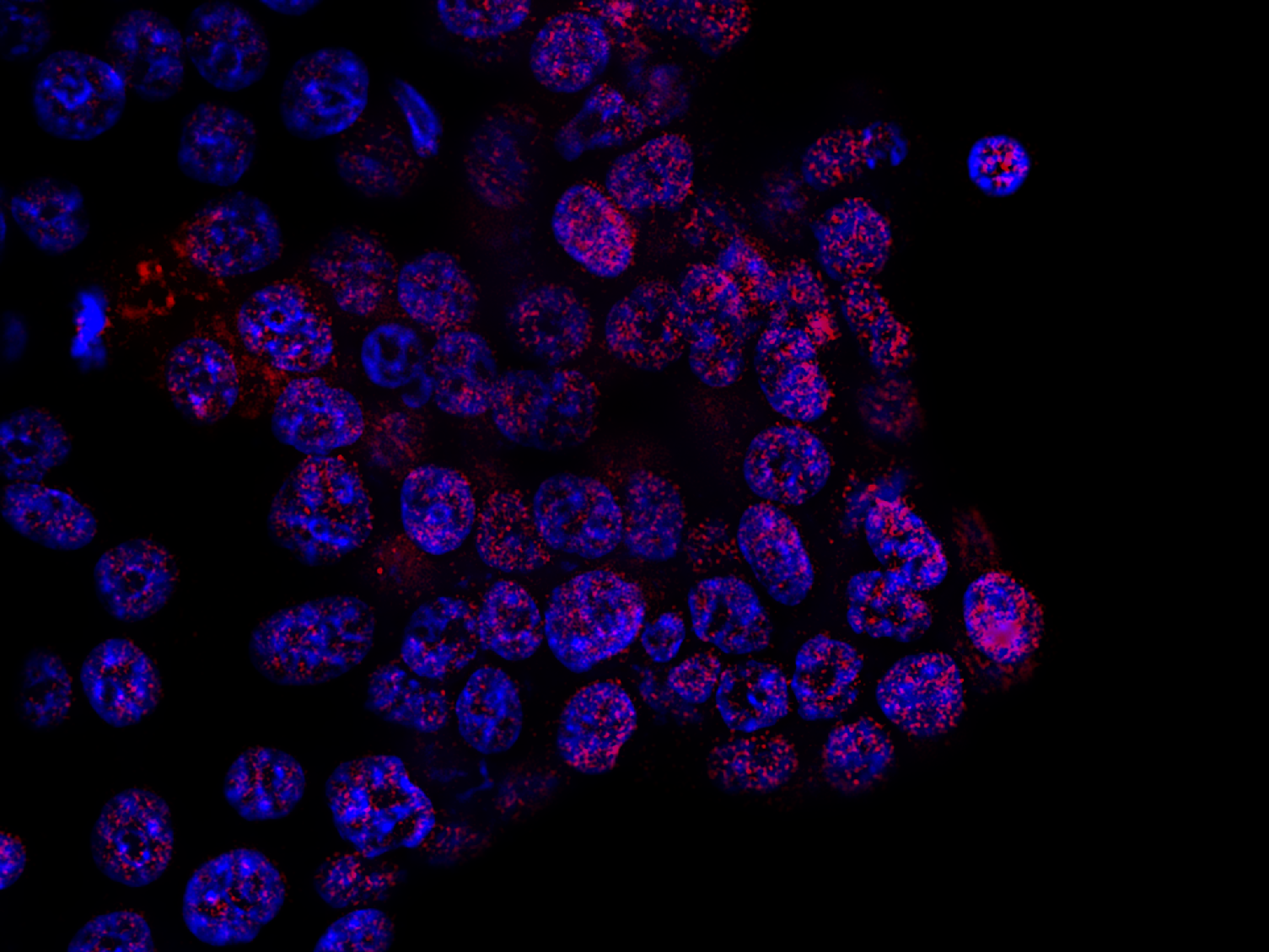

Supplement: Supplementary file 3 — Source data Fig. 2 [file 44319_2025_484_MOESM3_ESM.zip › Figure 2 Raw Data/2C/Figure 2C NuMA PLA Palmostatin HCT116 Interphase Representative Image.TIF]

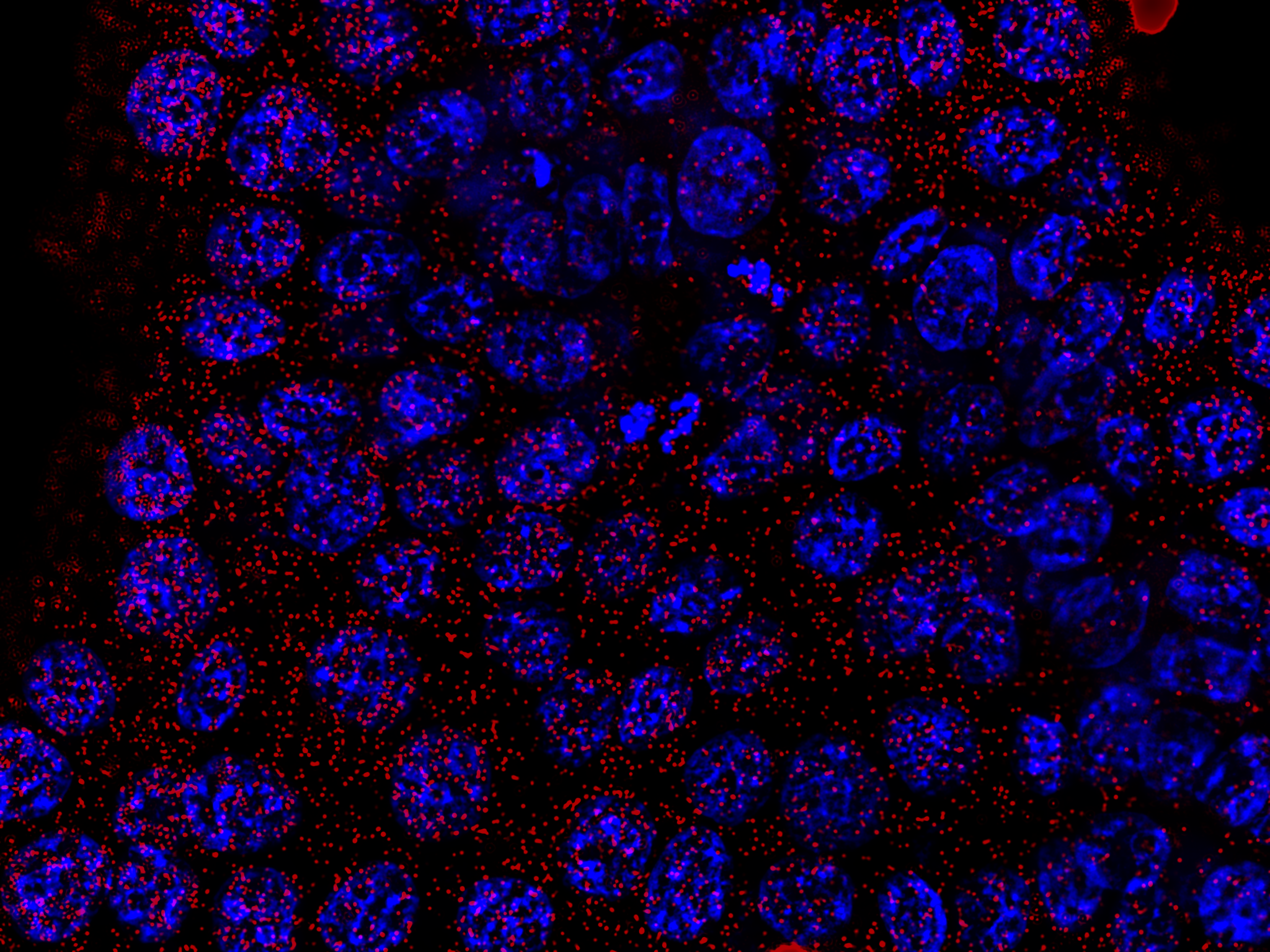

Supplement: Supplementary file 3 — Source data Fig. 2 [file 44319_2025_484_MOESM3_ESM.zip › Figure 2 Raw Data/2C/Figure 2C NuMA PLA Wnt-C59 HCT116 Interphase and Mitotic Representative Images.TIF]

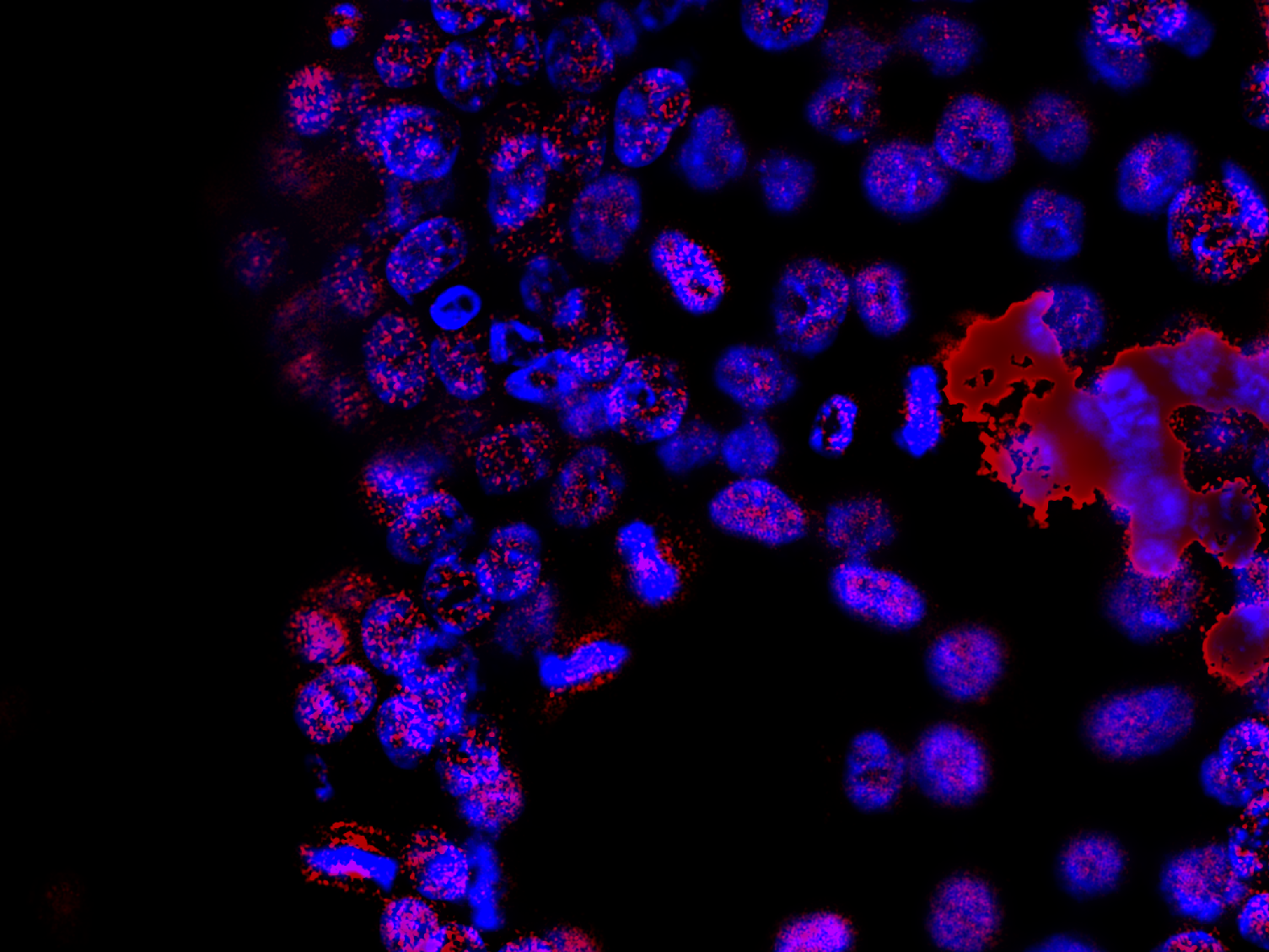

Supplement: Supplementary file 3 — Source data Fig. 2 [file 44319_2025_484_MOESM3_ESM.zip › Figure 2 Raw Data/2C/Figure 2C NuMA PLA Palmostatin HCT116 Mitotic Representative Image.TIF]

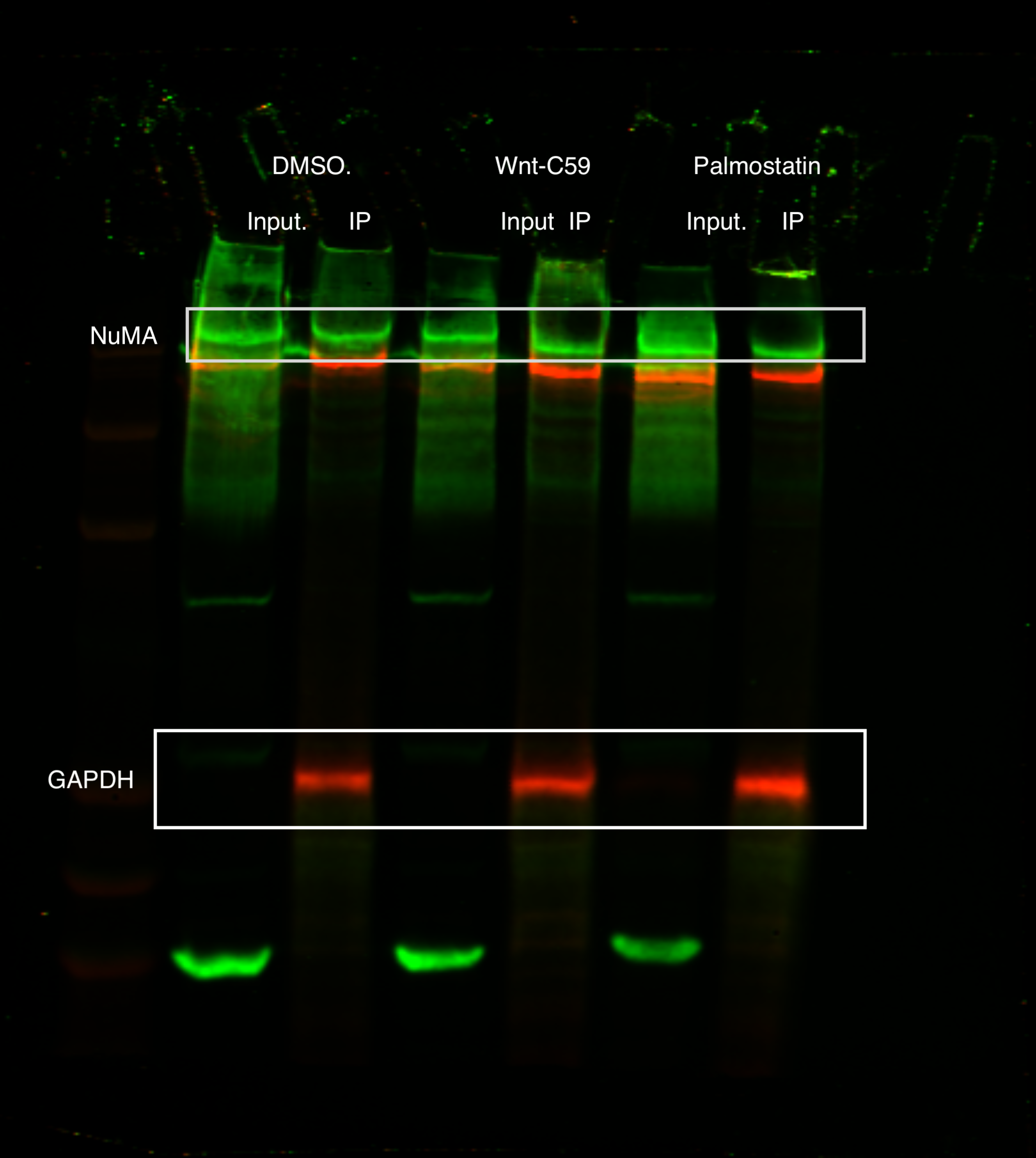

Supplement: Supplementary file 3 — Source data Fig. 2 [file 44319_2025_484_MOESM3_ESM.zip › Figure 2 Raw Data/2A/Figure 2A NuMA-KPNA2 CoIP NuMA and GAPDH.tif]

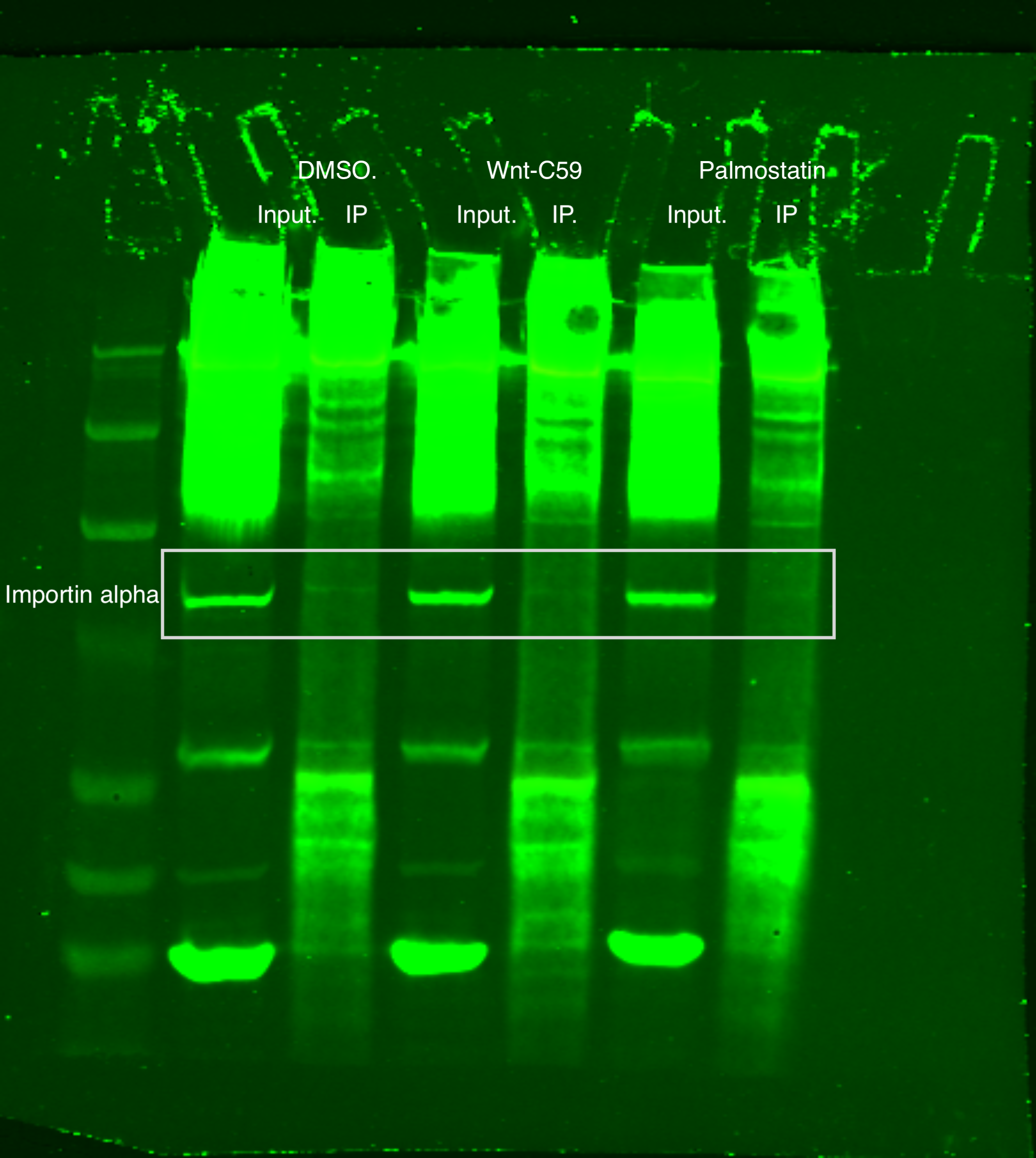

Supplement: Supplementary file 3 — Source data Fig. 2 [file 44319_2025_484_MOESM3_ESM.zip › Figure 2 Raw Data/2A/Figure 2A NuMA-KPNA2 CoIP Increased Exposure for KPNA2.tif]

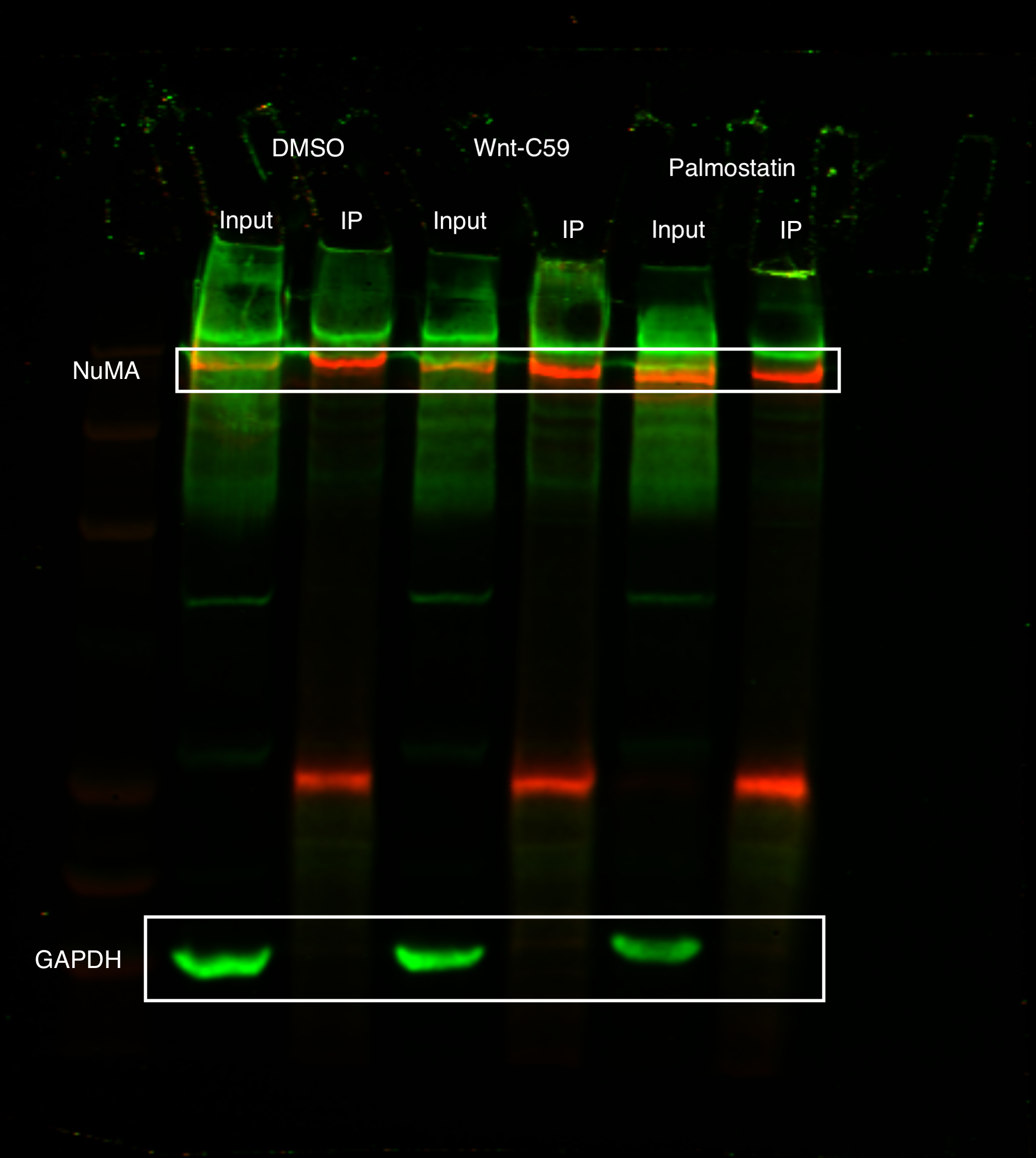

Supplement: Supplementary file 3 — Source data Fig. 2 [file 44319_2025_484_MOESM3_ESM.zip › Figure 2 Raw Data/2A/Figure 2A NuMA-KPNA2 CoIP NuMA and GAPDH corrected.tif]

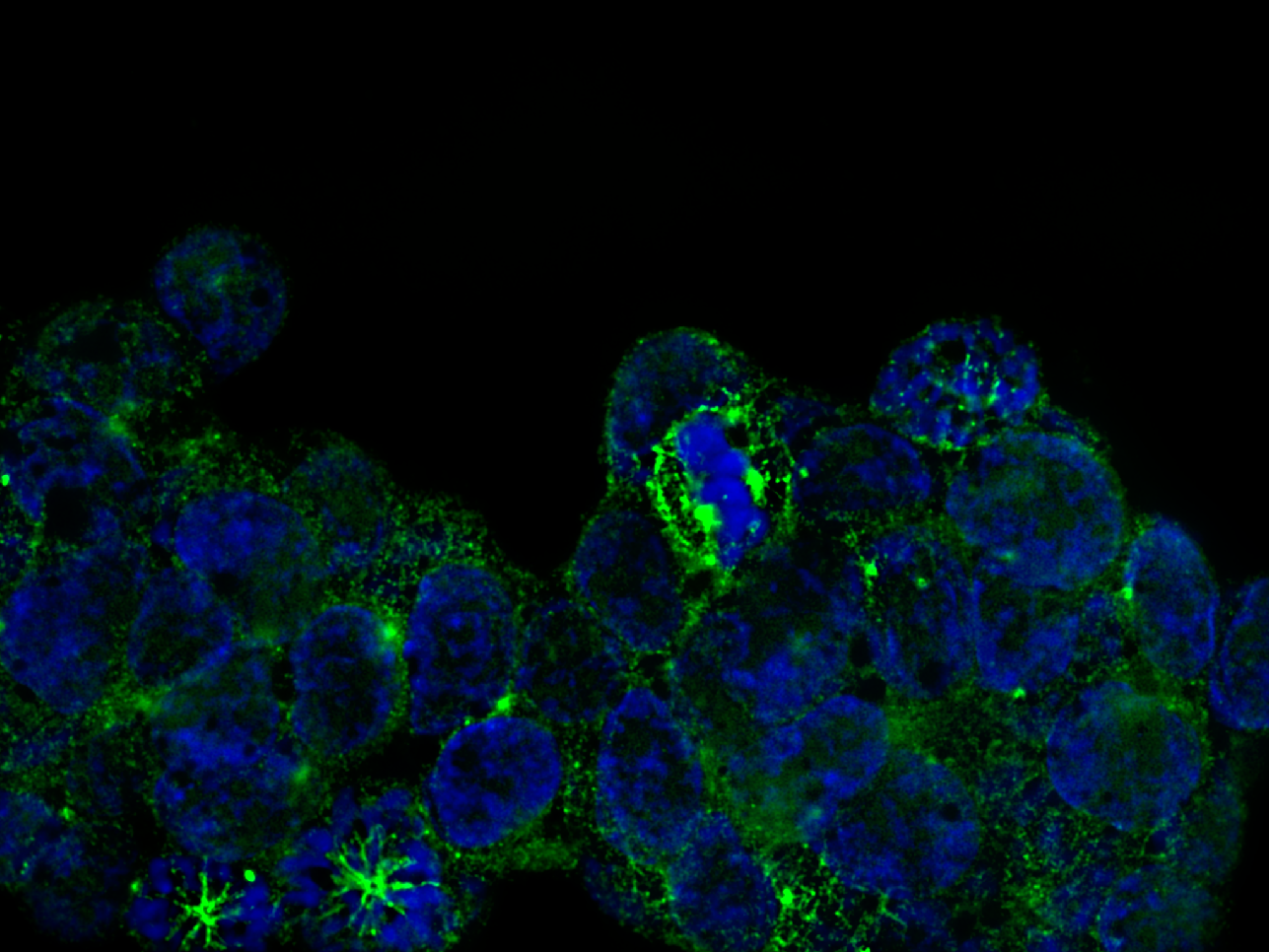

Supplement: Supplementary file 4 — Source data Fig. 3 [file 44319_2025_484_MOESM4_ESM.zip › Figure 3 Raw Data/3E/Figure 3E DMSO Representative Image.TIF]

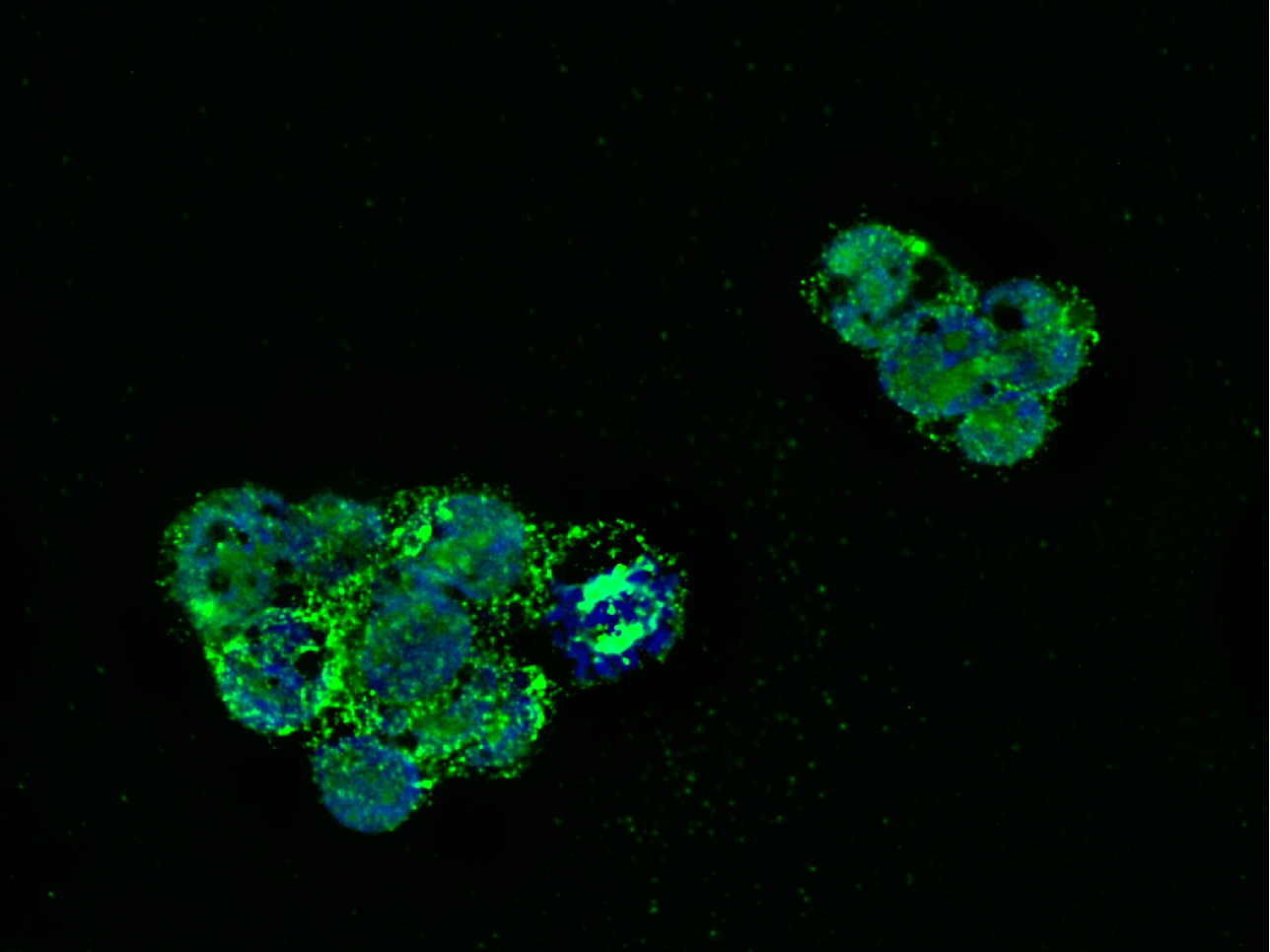

Supplement: Supplementary file 4 — Source data Fig. 3 [file 44319_2025_484_MOESM4_ESM.zip › Figure 3 Raw Data/3E/Figure 3E Palmostatin Representative Image.TIF]

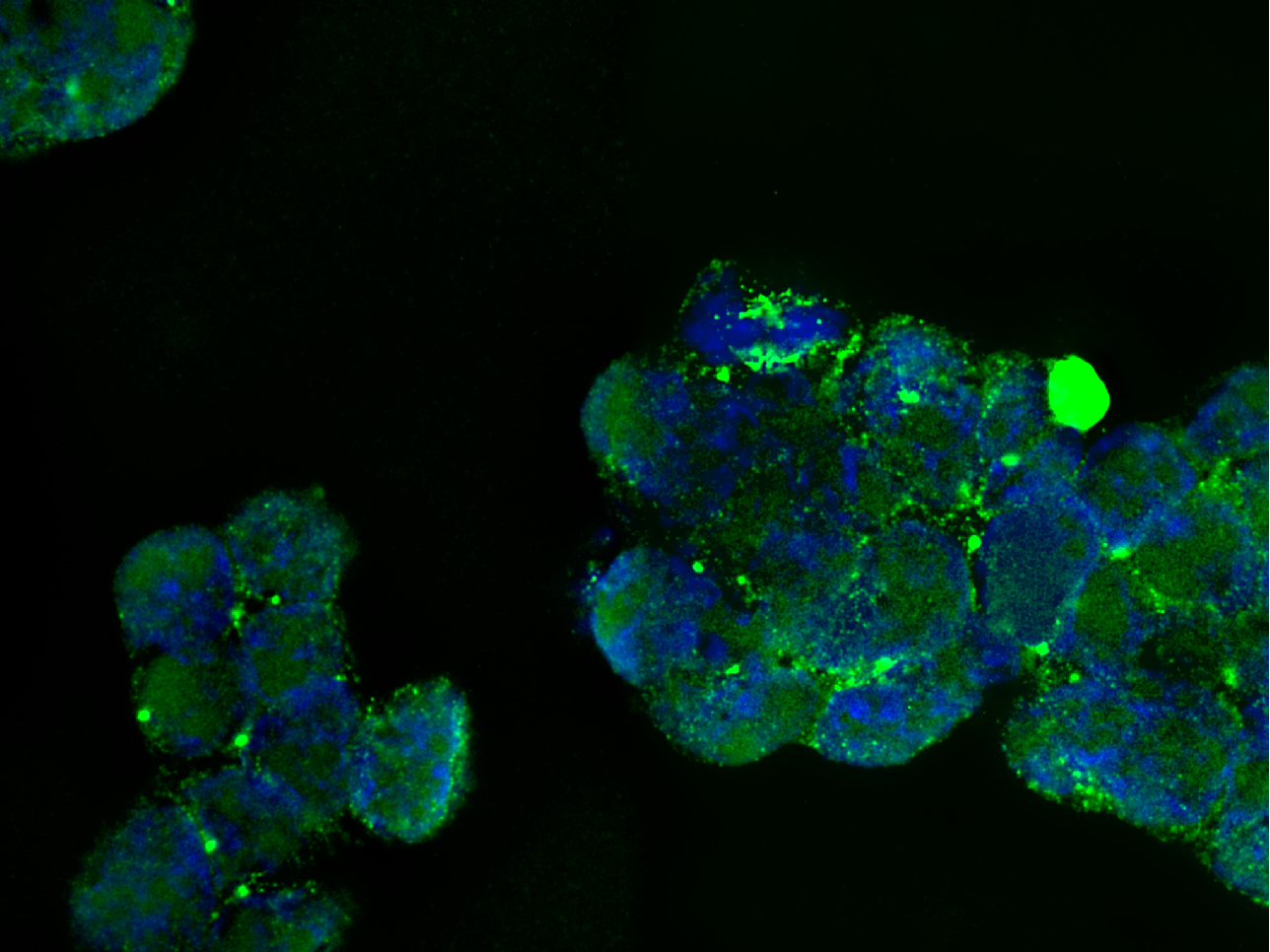

Supplement: Supplementary file 4 — Source data Fig. 3 [file 44319_2025_484_MOESM4_ESM.zip › Figure 3 Raw Data/3E/Figure 3E Wnt-C59 Representative Image.TIF]

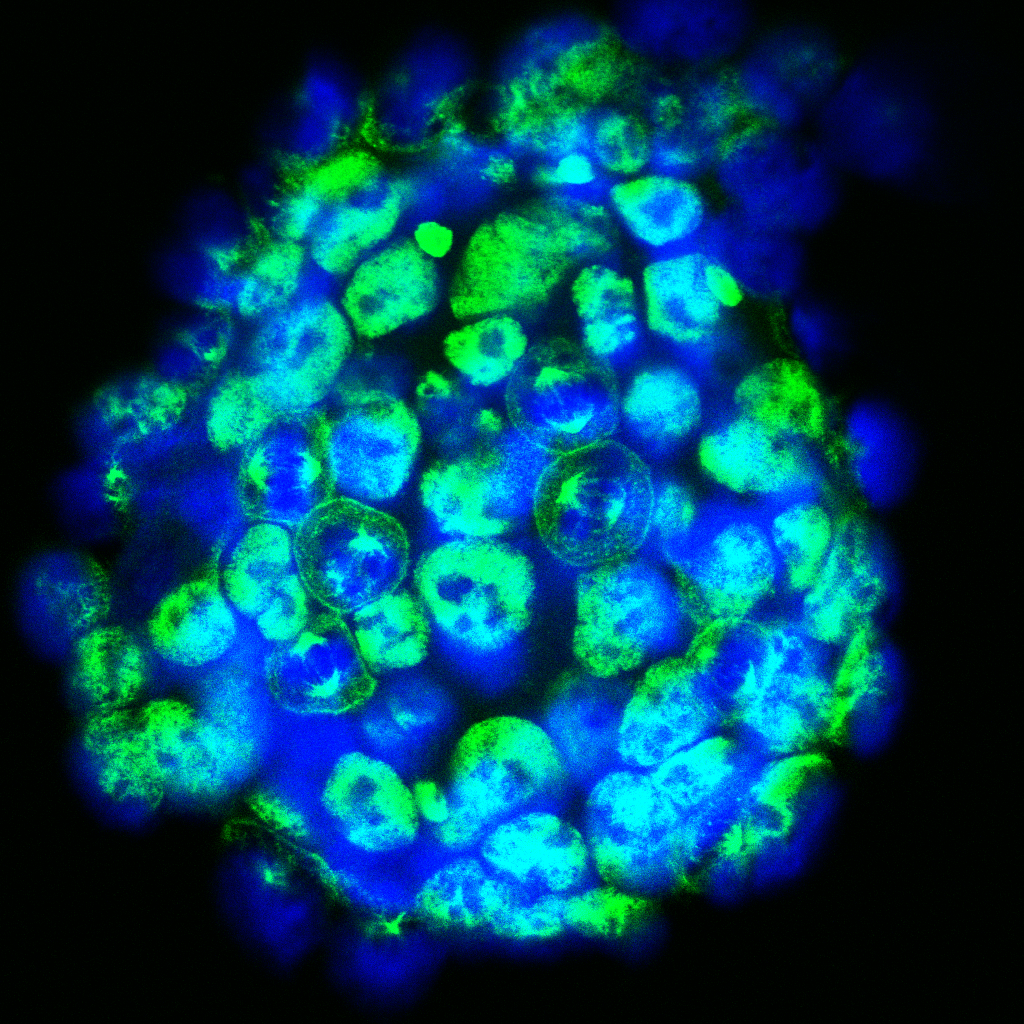

Supplement: Supplementary file 4 — Source data Fig. 3 [file 44319_2025_484_MOESM4_ESM.zip › Figure 3 Raw Data/3A/Figure 3A DMSO Representative Image.tif]

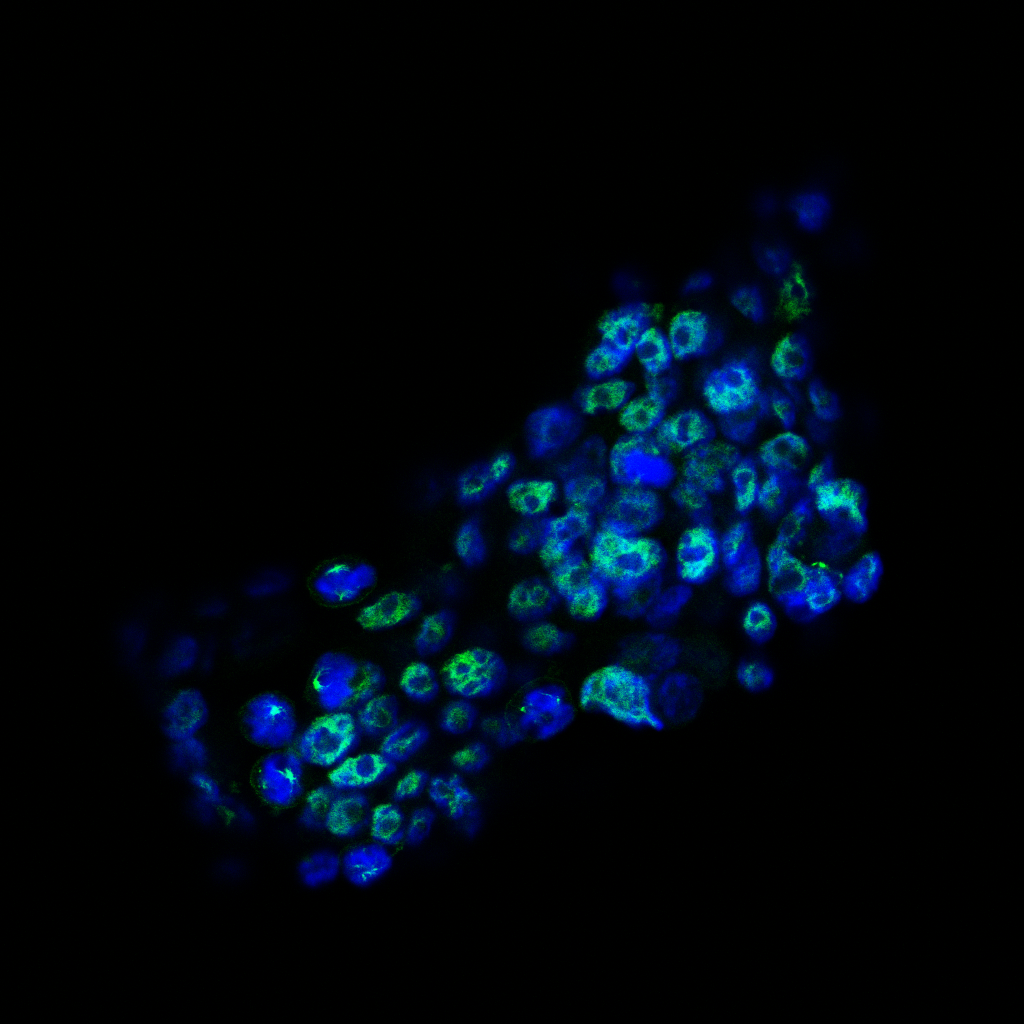

Supplement: Supplementary file 4 — Source data Fig. 3 [file 44319_2025_484_MOESM4_ESM.zip › Figure 3 Raw Data/3A/Figure 3A Importazole Representative Image.tif]

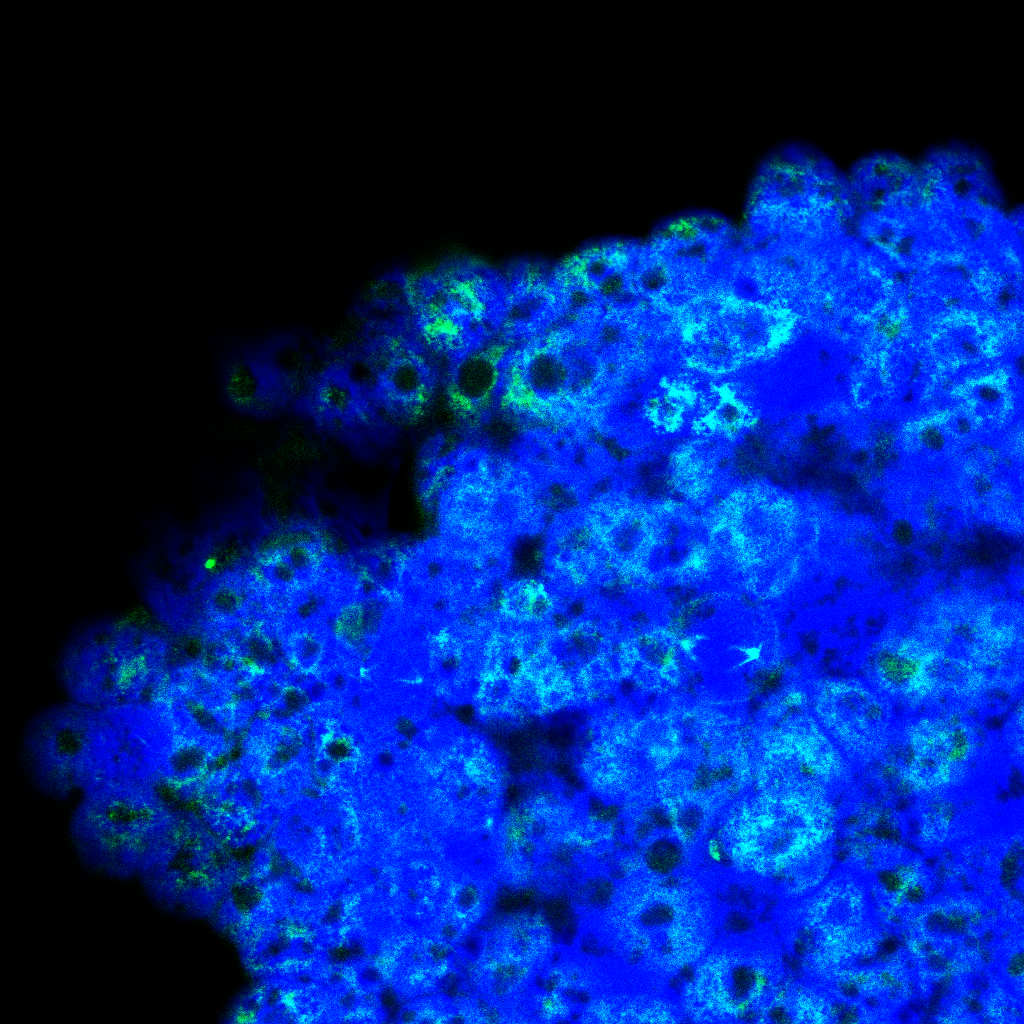

Supplement: Supplementary file 4 — Source data Fig. 3 [file 44319_2025_484_MOESM4_ESM.zip › Figure 3 Raw Data/3A/Figure 3A Ivermectin Representative Image.tif]

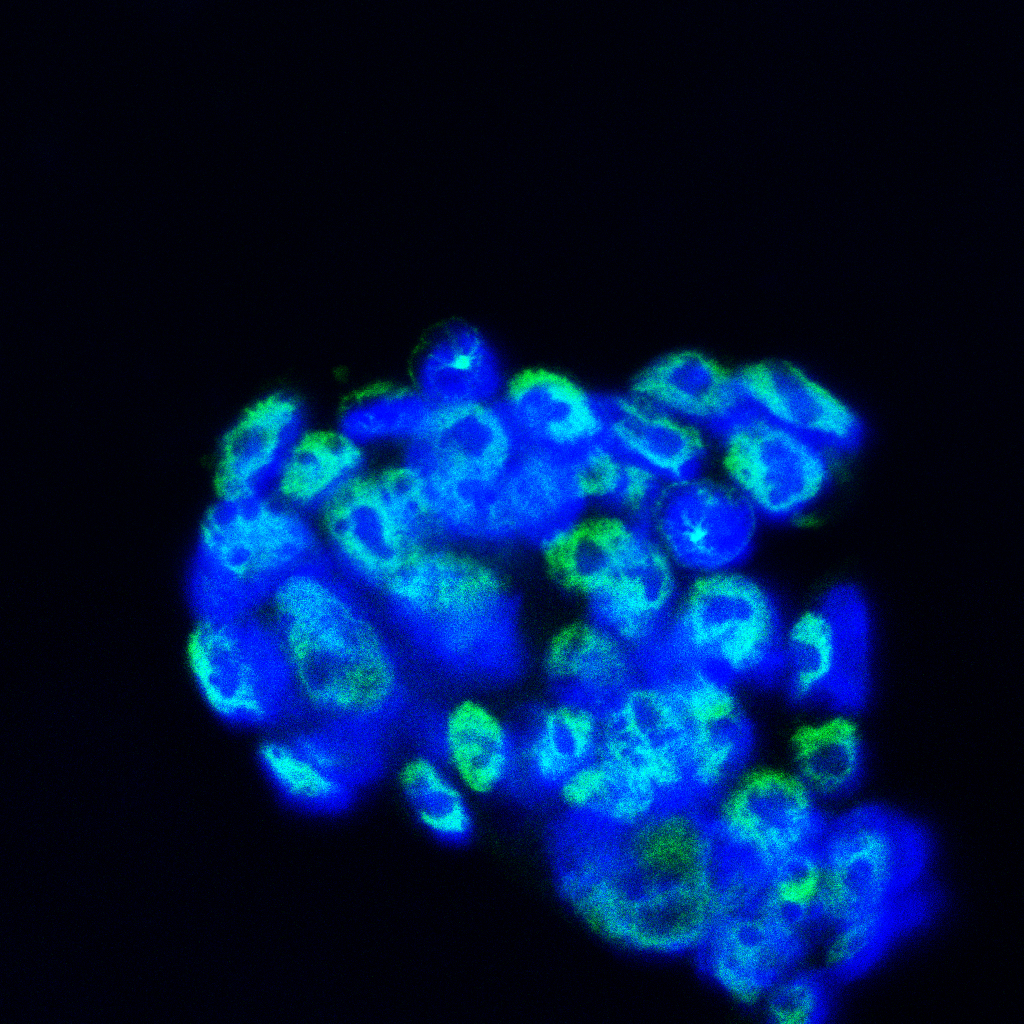

Supplement: Supplementary file 4 — Source data Fig. 3 [file 44319_2025_484_MOESM4_ESM.zip › Figure 3 Raw Data/3A/Figure 3A Palmostatin Representative Image.tif]

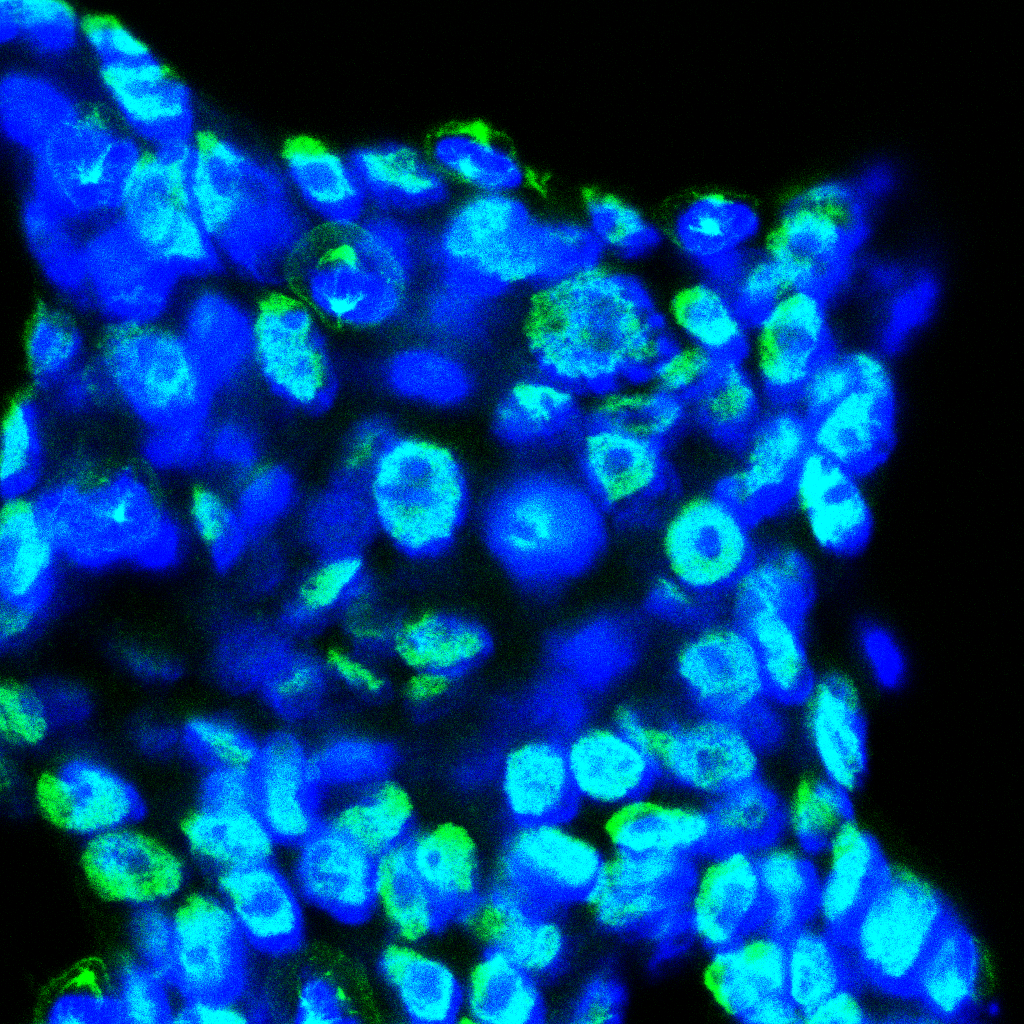

Supplement: Supplementary file 4 — Source data Fig. 3 [file 44319_2025_484_MOESM4_ESM.zip › Figure 3 Raw Data/3A/Figure 3A Wnt-C59 Representative Image.tif]

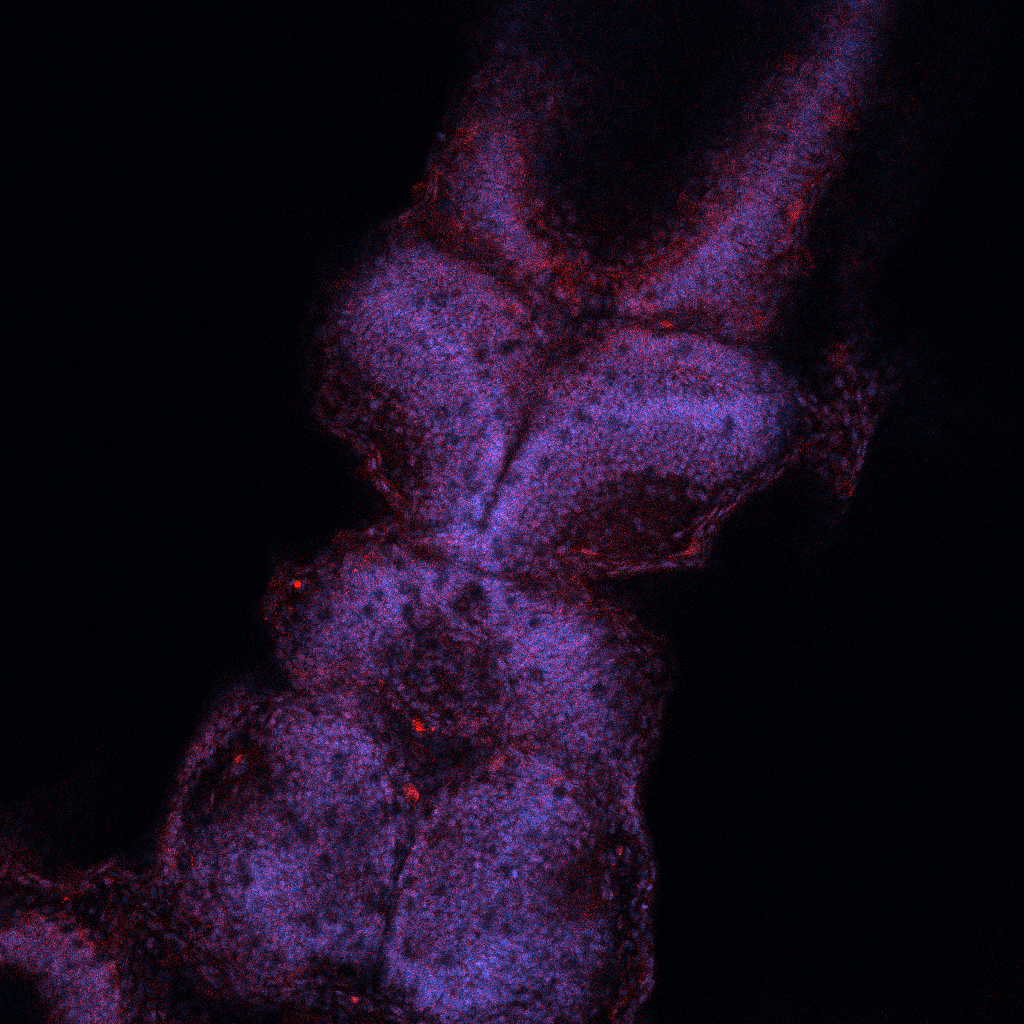

Supplement: Supplementary file 5 — Source data Fig. 4 [file 44319_2025_484_MOESM5_ESM.zip › Figure 4 Raw Data/4C/Figure 4C DMSO brain nestin 10X representative image.TIF]

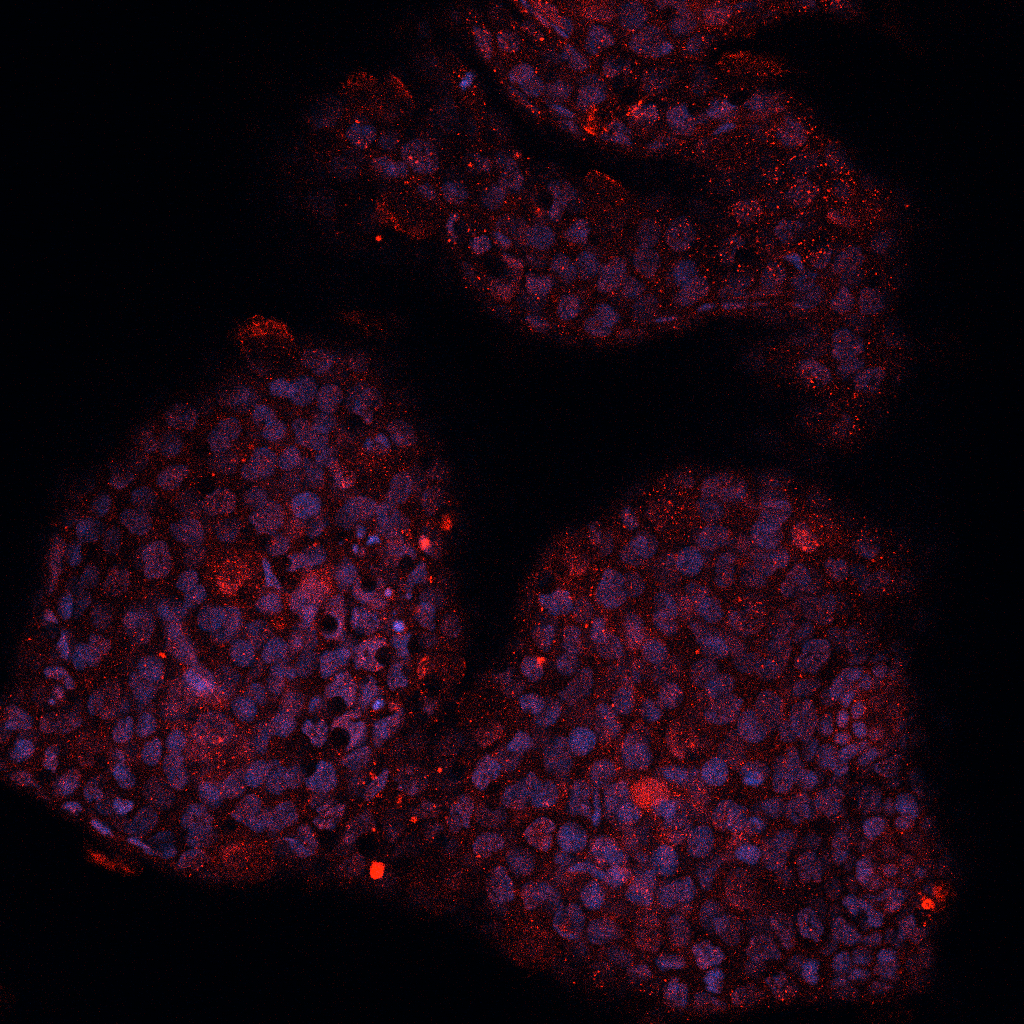

Supplement: Supplementary file 5 — Source data Fig. 4 [file 44319_2025_484_MOESM5_ESM.zip › Figure 4 Raw Data/4C/Figure 4C DMSO forebrain nestin 20X representative image.TIF]

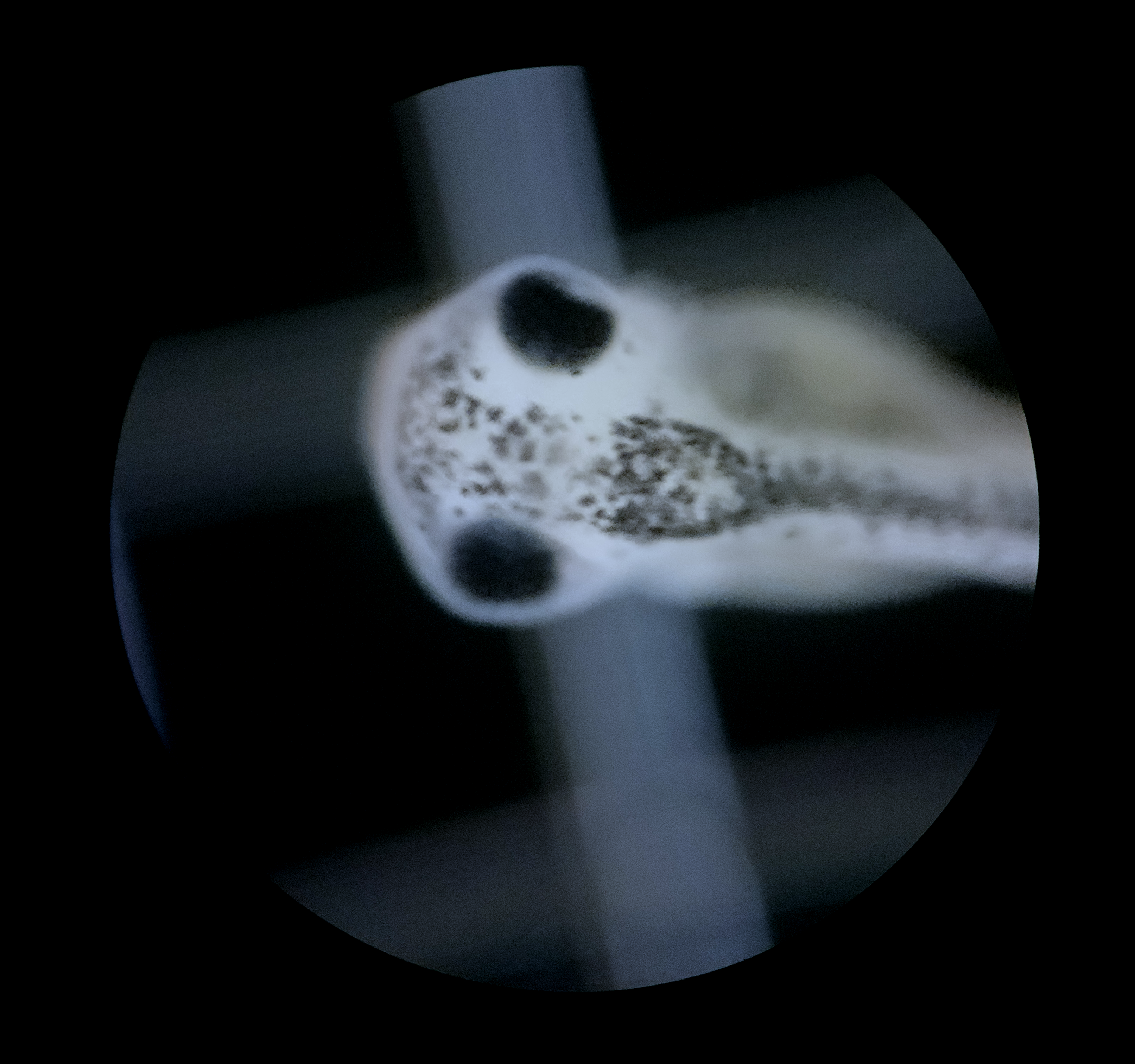

Supplement: Supplementary file 5 — Source data Fig. 4 [file 44319_2025_484_MOESM5_ESM.zip › Figure 4 Raw Data/4A/Figure 4A DMSO representative image.tiff]

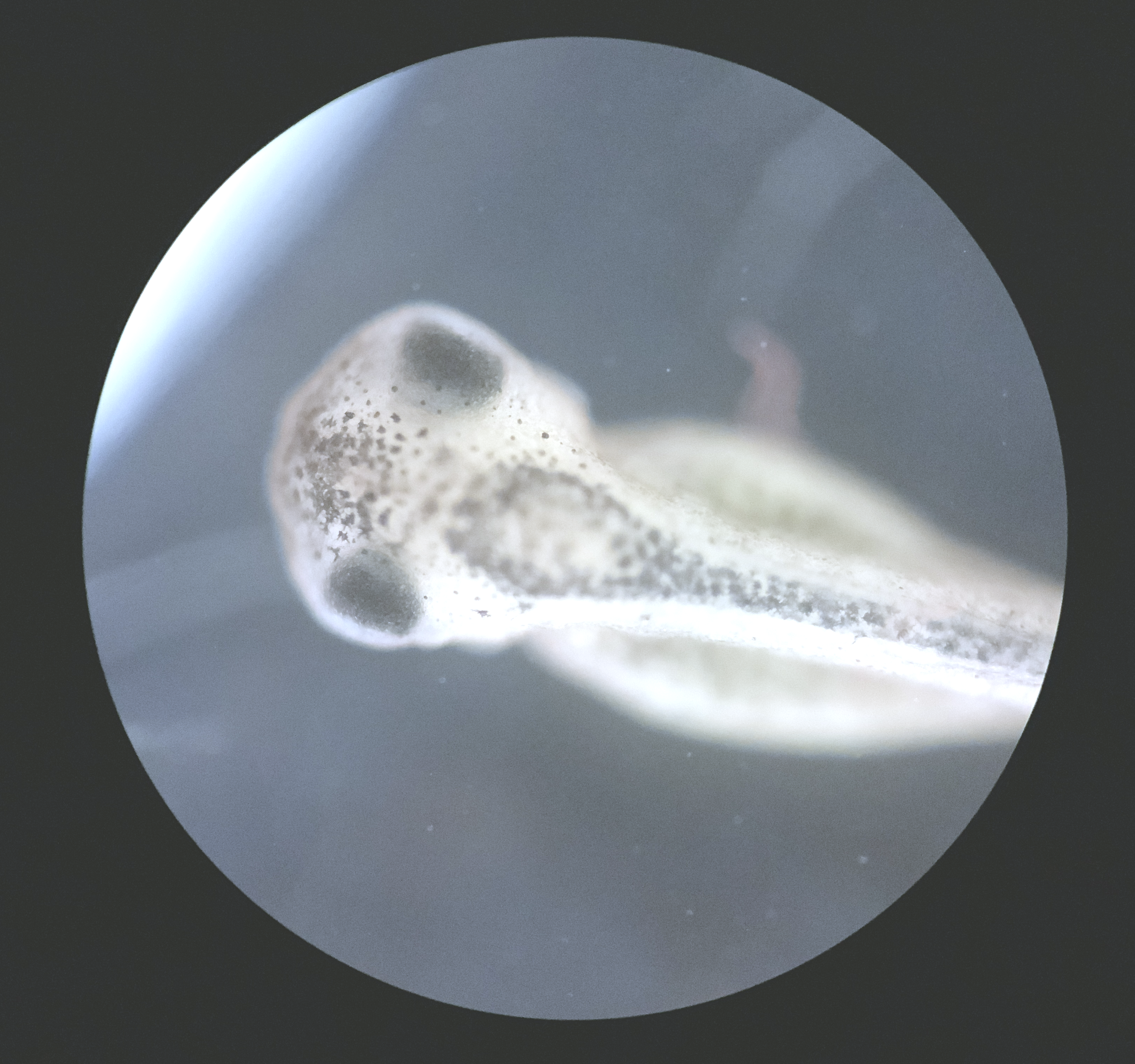

Supplement: Supplementary file 5 — Source data Fig. 4 [file 44319_2025_484_MOESM5_ESM.zip › Figure 4 Raw Data/4A/Figure 4A Palmostatin Representative Image.tiff]

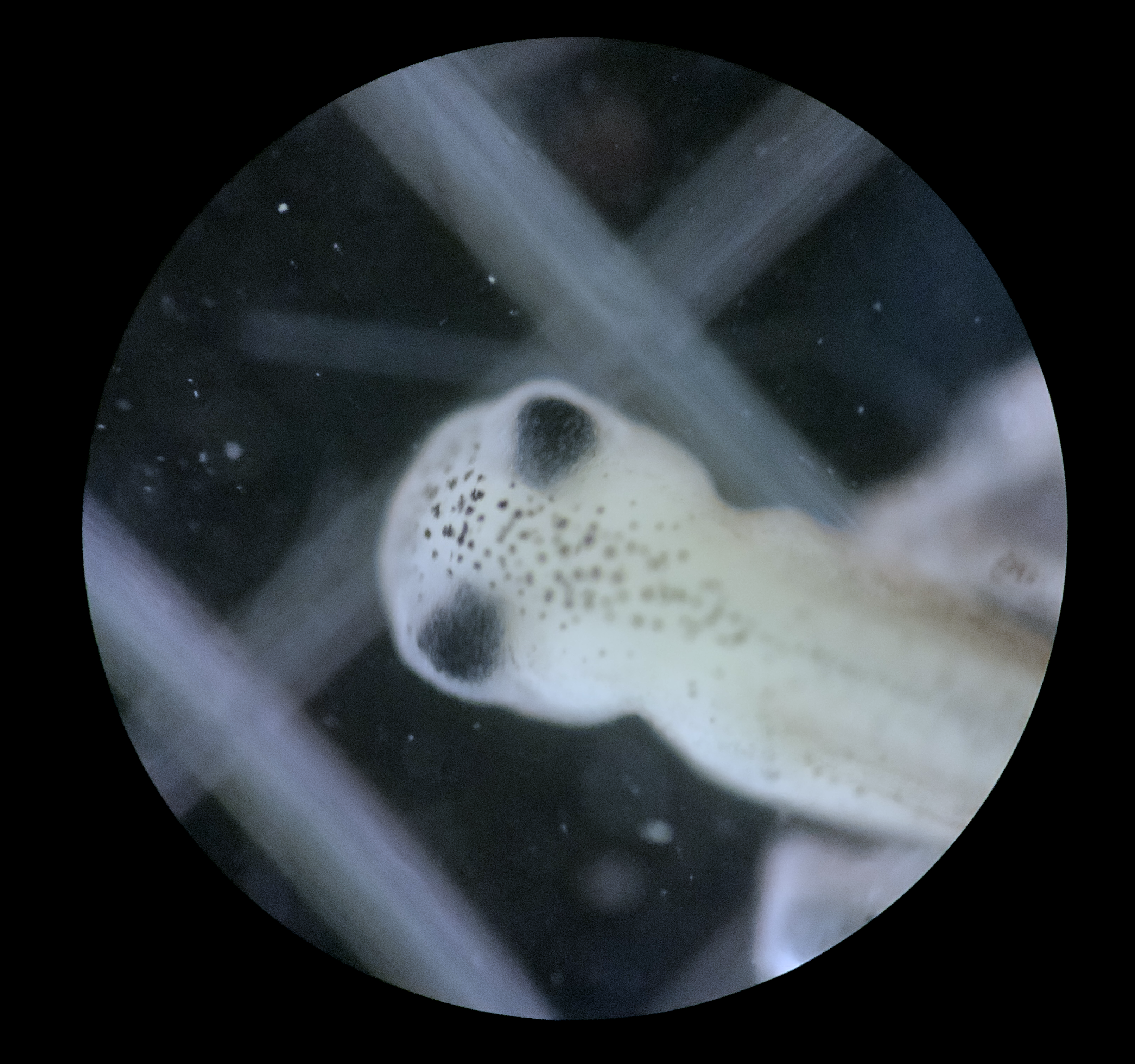

Supplement: Supplementary file 5 — Source data Fig. 4 [file 44319_2025_484_MOESM5_ESM.zip › Figure 4 Raw Data/4A/Figure 4A Wnt-C59 Representative Image.tiff]

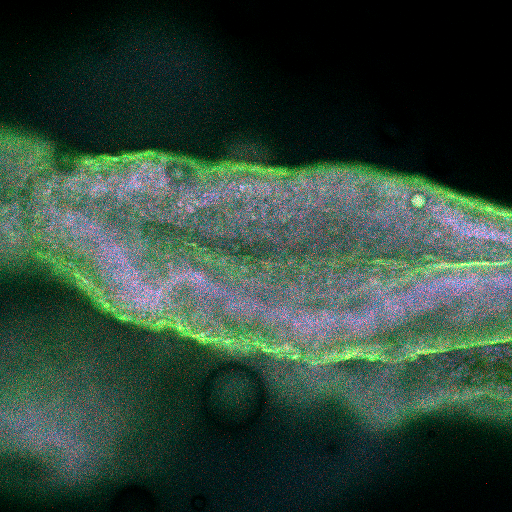

Supplement: Supplementary file 6 — Source data Fig. 5 [file 44319_2025_484_MOESM6_ESM.zip › Figure 5 Raw Data/5C/Figure 5C mCherry CaaX Representative Image.tif]

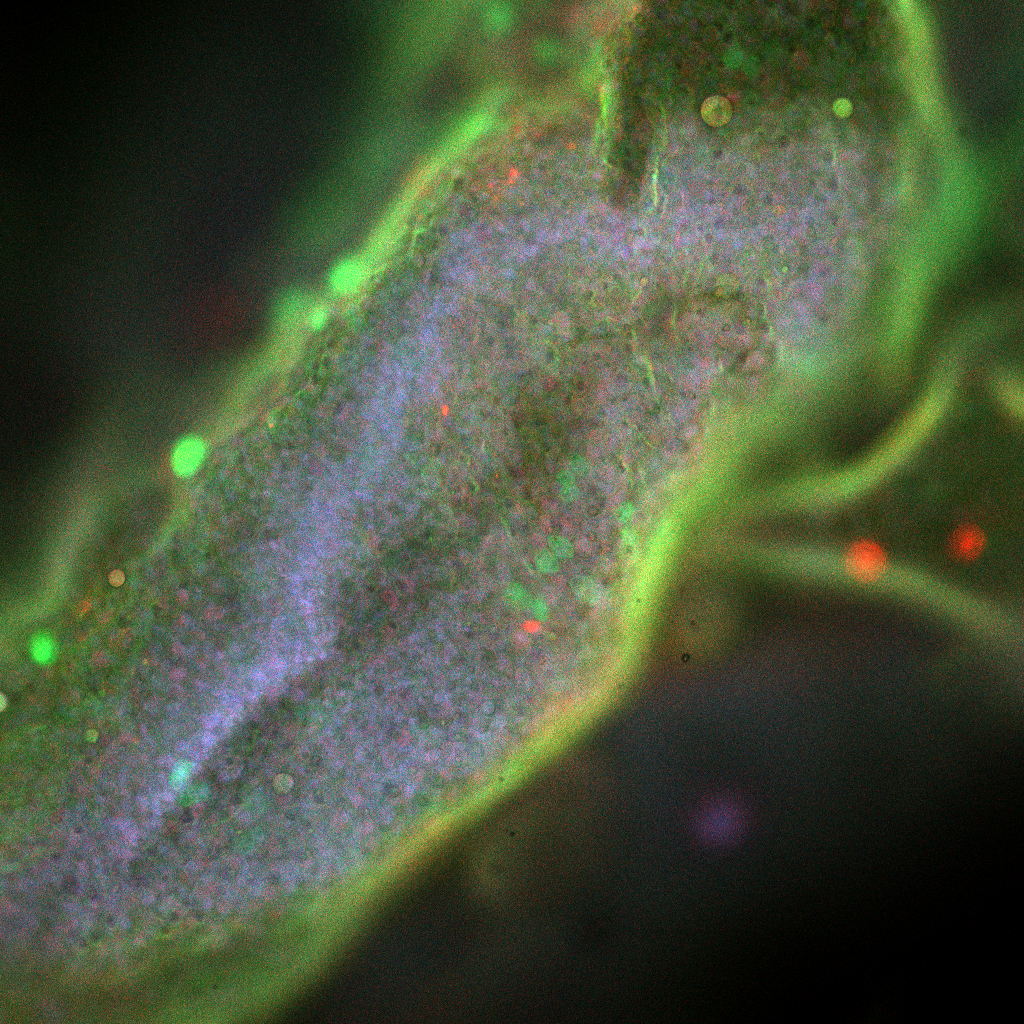

Supplement: Supplementary file 6 — Source data Fig. 5 [file 44319_2025_484_MOESM6_ESM.zip › Figure 5 Raw Data/5B/Figure 5B importin alpha mCherry CaaX Representative Image.tif]

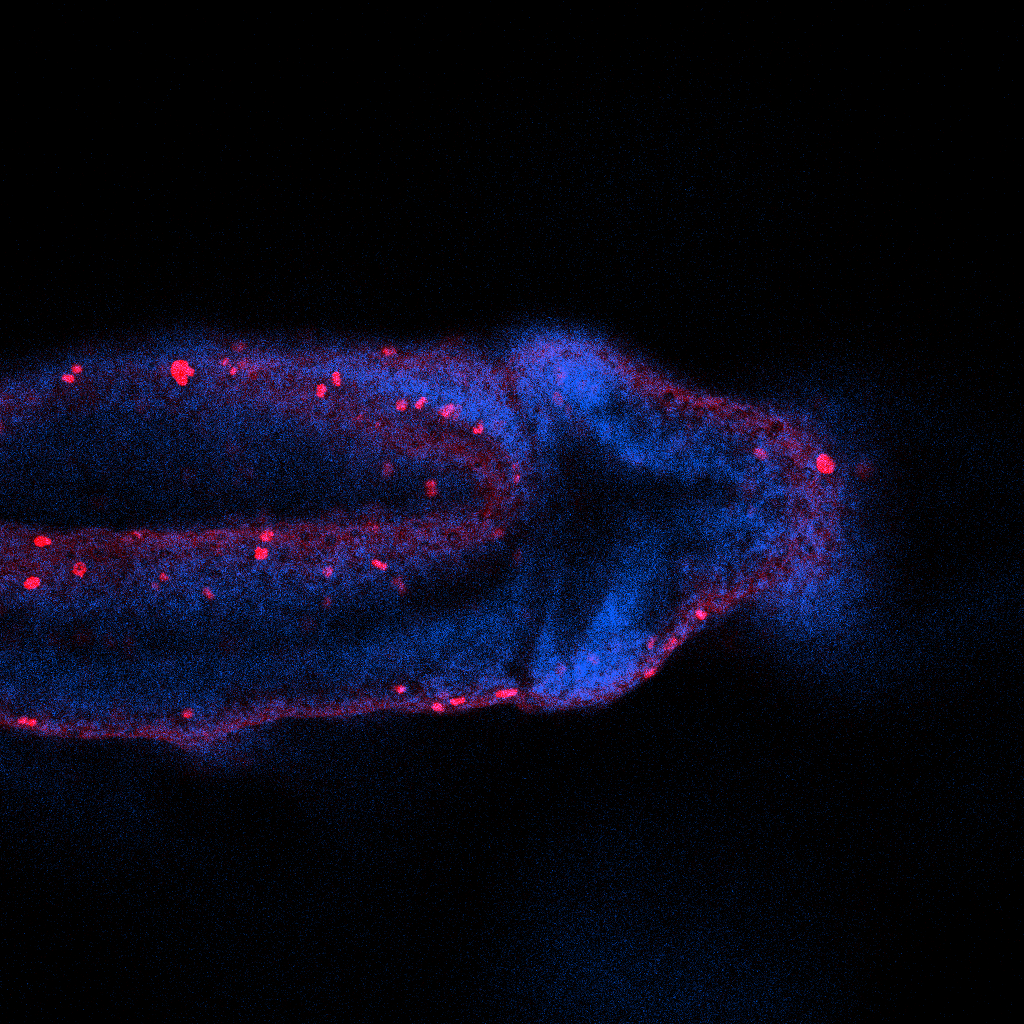

Supplement: Supplementary file 6 — Source data Fig. 5 [file 44319_2025_484_MOESM6_ESM.zip › Figure 5 Raw Data/5A/Figure 5A DMSO Representative Image.TIF]

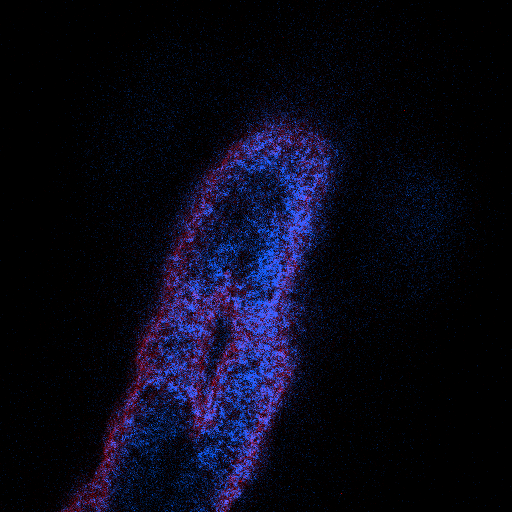

Supplement: Supplementary file 6 — Source data Fig. 5 [file 44319_2025_484_MOESM6_ESM.zip › Figure 5 Raw Data/5A/Figure 5A Wnt-C59 Representative Image.TIF]
