# Supplementary material for: Palmitoylated importin α regulates mitotic spindle orientation through interaction with NuMA
Source: EMBO Rep. 2025 May 27;26(13):3280–304. doi: 10.1038/s44319-025-00484-8 (PMC12238373; doi:10.1038/s44319-025-00484-8)
Supplement: Supplementary file 7 — Expanded View Figures [file 44319_2025_484_MOESM7_ESM.pdf]

Expanded View Figures

A

Position	Peptide	Score
133	KFVSFLGRTDCSPIQFESAWA	0.288
223	LAVPDMSSLACGYLRNLTWTL	0.6991
237	RNLTWTLSNLCRNKNPAPPID	0.6514
272	HDDPEVLADTCWAISYLTDGP	0.1746
419	TVEQIVYLVHCGIIEPLMNLL	0.1964
467	TEKLSIMIEECGGLDKIEALQ	0.7908

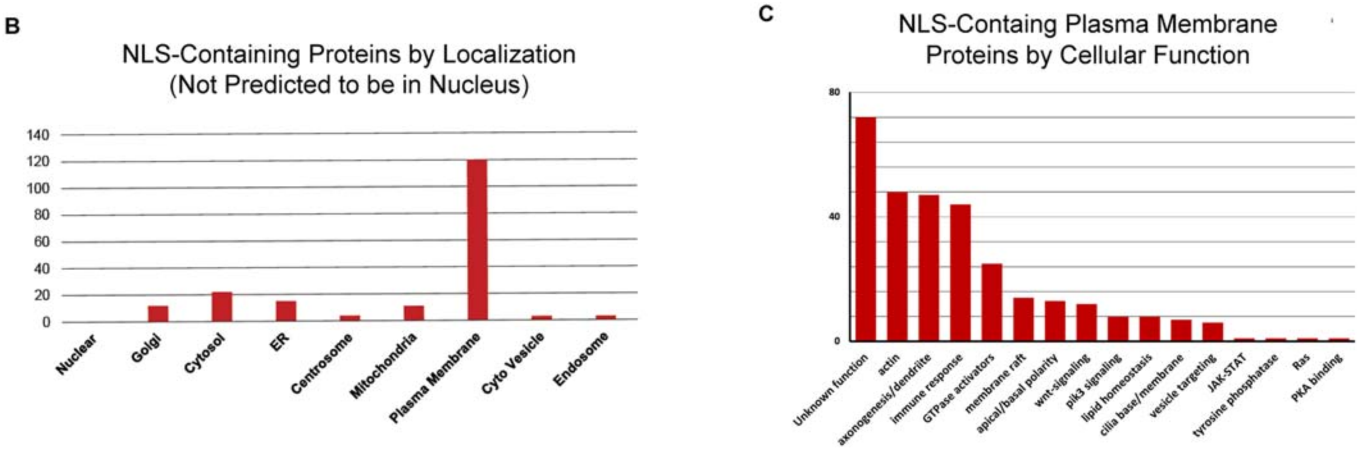

Figure EV1. Proteome screens confirm palmitoylation of human importin α-1 and enrichment of NLS containing proteins by cellular localization and function.

(**A**) Prediction of palmitoylated cysteine residues in Human Importin α-1 (KPNA2) by GPS-Palm. Prediction score is on a 0-1 scale with 0 representing low confidence of palmitoylation and 1 representing high confidence of palmitoylation. Three highest confidence residues are highlighted in green. (**B**) Cellular localization of NLS containing proteins not predicted to be in the nucleus. (**C**) Cellular functions of NLS containing proteins found at the PM sorted by gene ontology terms.

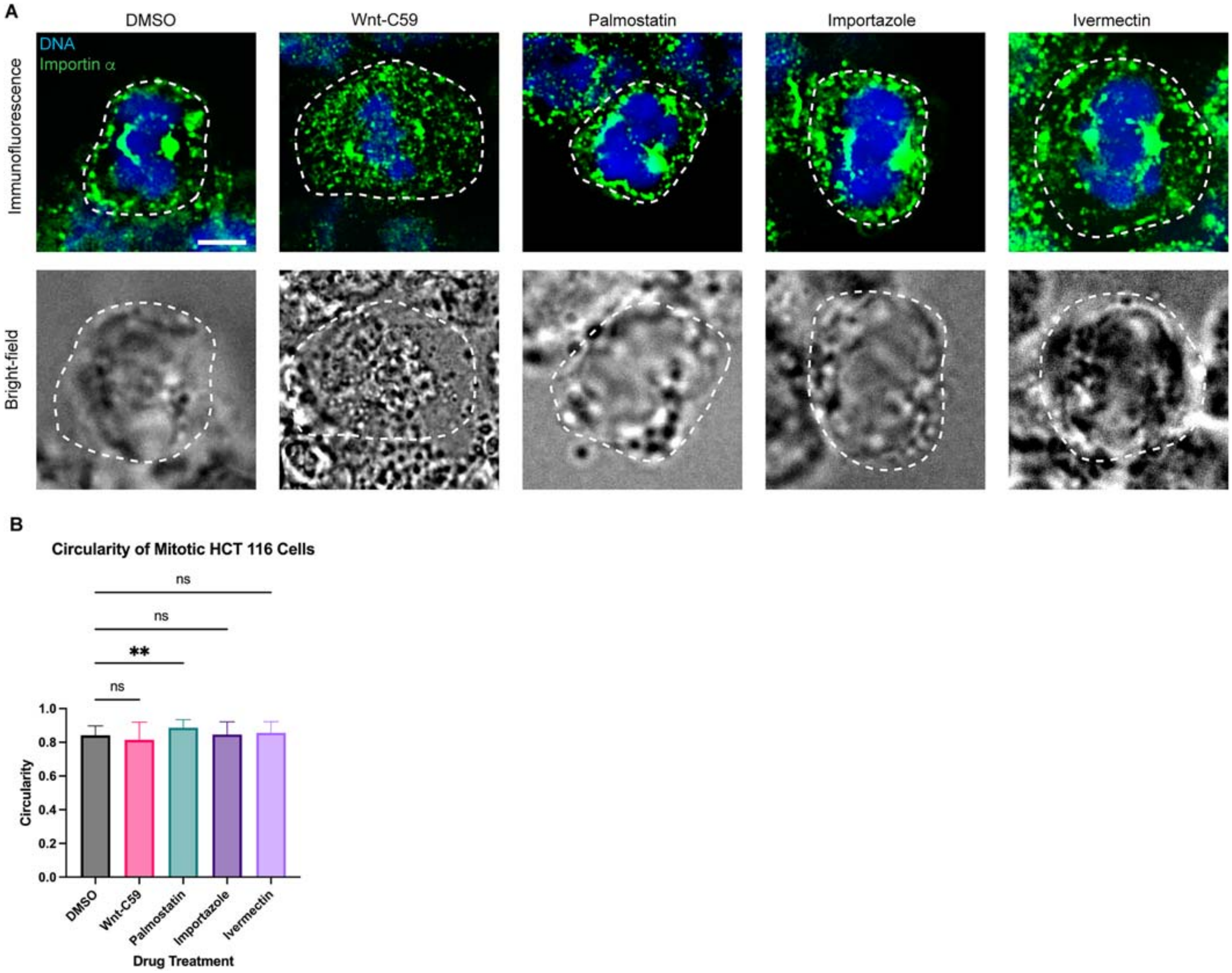

Figure EV2. KPNA2 cellular localization boundary determination.

(**A**) Representative immunofluorescence and bright-field images for cells used in Fig. 1A to determine KPNA2 localization and cell boundary determination for each drug treatment. Scale bar = 5 µm. (**B**) Quantification of the circularity of metaphase-arrested HCT116 cells treated with DMSO, 10 µM Wnt-C59, 50 µM palmostatin, 40 µM importazole or 25 µM ivermectin for 1 h. Mean +/− SEM $n = 60$, ** $p = 0.0031$ determined by Student's t-test.

 © The Author(s)

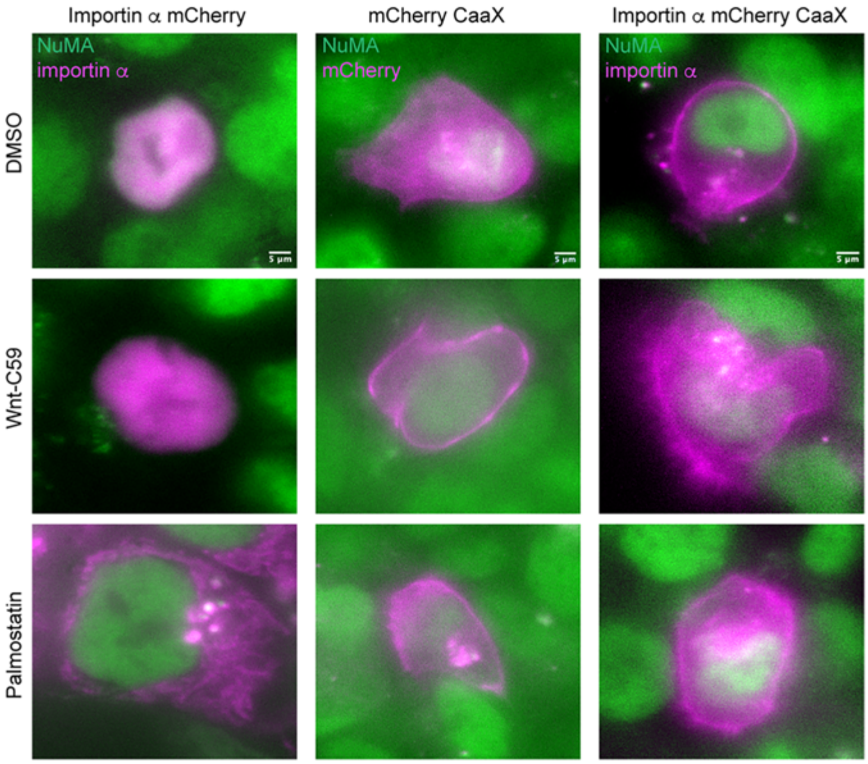

Figure EV3. CaaX modified importin α localizes to the plasma membrane independent of palmitoylation.

Immunofluorescent images of HCT116 cells transfected with importin α-mCherry, mCherry-CaaX or importin α-mCherry-CaaX treated with DMSO, Wnt-C59 or palmostatin. Importin α-mCherry-CaaX localizes to the plasma membrane in all drug treatments. Scale bar = 5 µm.

© The Author(s)

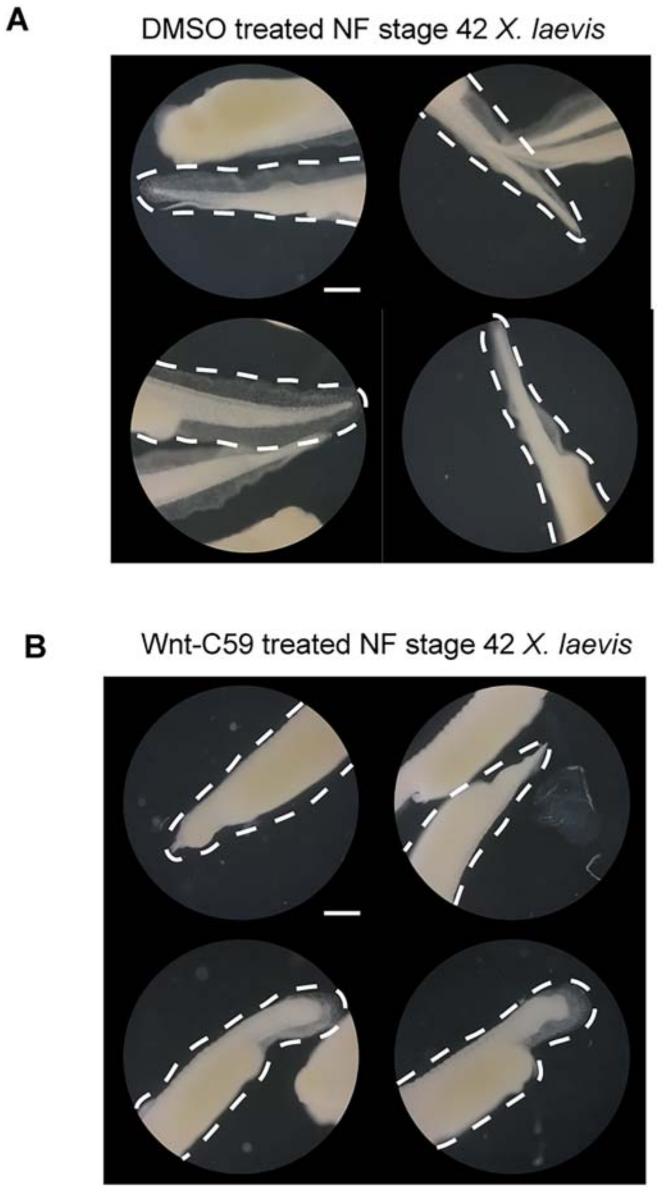

Figure EV4. Wnt-C59 treated *X. laevis* tadpoles exhibit shortened tail length.

(**A**) Brightfield images of NF Stage 42 *X. laevis* tadpole tails treated with DMSO from 24 to 72 hpf. DMSO treated tadpoles exhibit normal development and typical length tails. Scale bar = 500 μm. White dashed line indicates examined tadpole tail for each image. (**B**) Brightfield images of NF Stage 42 *X. laevis* tadpole tails treated with Wnt-C59 from 24 to 72 hpf. Wnt-C59 treated tadpoles exhibit abnormal development, shortened tails, and abnormally shaped tails. Scale bar = 500 μm. White dashed line indicates examined tadpole tail for each image.

 © The Author(s)

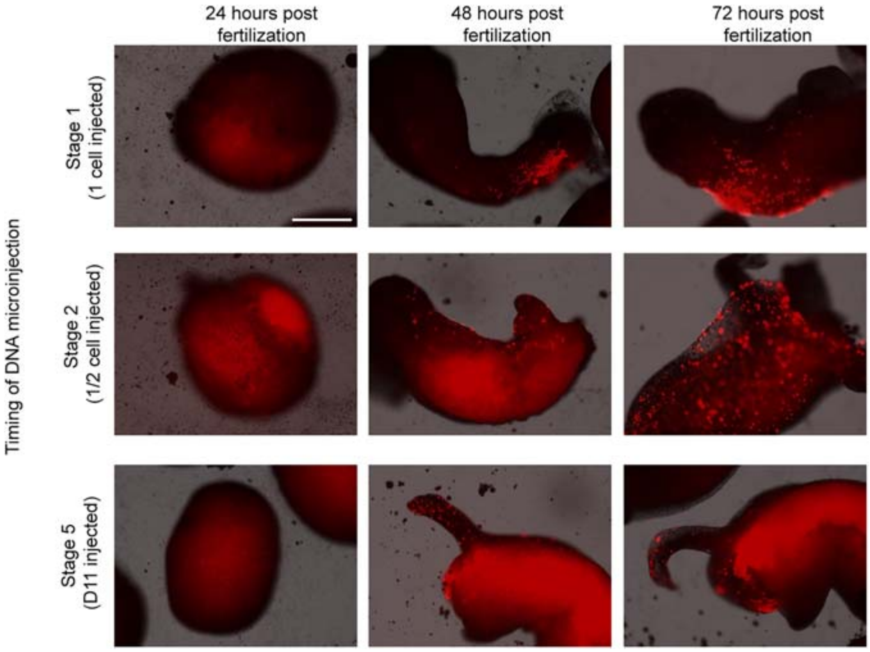

Figure EV5. Importin α overexpression produces severe developmental defects in *X. laevis* embryos.

Immunofluorescent images of *X. laevis* embryos co-injected with importin α-mCherry-CaaX pcDNA4TO and pcDNA6TR at 24, 48, and 72 h post fertilization. Embryos were injected at either the 1 cell, 2 cell (injected into 1 of 2 cells), or 16 cell stage (injected into the D11 blastomere). All 1 and 2 cell injected embryos exhibit high mortality and severe developmental defects. D11 injected embryos had better survivability though some embryos still exhibited defects. Scale bar = 500 μm.
